# Conserved interactions with stromal and immune cells coordinate de novo B cell lymphopoiesis in fetal intestines

**DOI:** 10.1172/jci.insight.192550

**Published:** 2025-09-02

**Authors:** Kimberly A. Carroll, Weihong Gu, Long Phan, Eduardo Gonzalez Santiago, Wenjia Wang, George C. Tseng, Liza Konnikova, Shruti Sharma

**Affiliations:** 1 Graduate School of Biomedical Sciences, Tufts University, Boston, Massachusetts, USA.; 2 Department of Pediatrics, Yale School of Medicine, New Haven, Connecticut, USA.; 3 Department of Biostatistics, University of Pittsburgh, Pittsburgh, Pennsylvania, USA.; 4 Department of Obstetrics, Gynecology and Reproductive Sciences,; 5 Department of Immunobiology,; 6 Program in Human and Translational Immunology,; 7 Program in Translational Biomedicine, and; 8 Center for Systems and Engineering Immunology, Yale School of Medicine, New Haven, Connecticut, USA.; 9 Department of Immunology, Tufts University School of Medicine, Boston, Massachusetts, USA.

**Keywords:** Development, Immunology, Adaptive immunity

## Abstract

Recent findings suggest that the small intestine (SI) is a potentially novel site for B cell lymphopoiesis during fetal and neonatal life. However, the unique and/or conserved features that enable B cell development at this site remain unclear. To investigate the molecular and cellular scaffolds for B cell lymphopoiesis in mouse and human fetal intestines, we leveraged single-cell RNA-Seq, in situ immunofluorescence, spatial transcriptomics, and high-dimensional spectral flow cytometry. We found that SI mesenchymal and stromal cells expressed higher levels of chemokines known to recruit common lymphoid progenitors. Importantly, local lymphatic endothelial cells expressed IL-7 and TSLP in proximity to IL-7R^+^ precursor B cells, likely promoting their differentiation in the SI. Notably, we found that fetal-derived lymphoid tissue inducer (LTi) cells were required for B cell development and localization in the SI, but not fetal liver. These findings identify a lymphoid tissue development–independent role for this immune cell in B cell development. Collectively, our data reveal a conserved intestinal B cell niche in mice and humans, challenging traditional models of lymphopoiesis. The identification of a requisite cellular/molecular scaffold for fetal B cell development allows future studies to test the importance of this de novo B cell lymphopoiesis to long-term immunity.

## Abstract 2

Our study describes an incredibly novel mechanism that shapes the local development of B-cell populations in fetal intestines. Critically, our study is based on seminal data in humans which we closely replicate and mechanistically dissect in relevant mouse-models.

## Graphical abstract

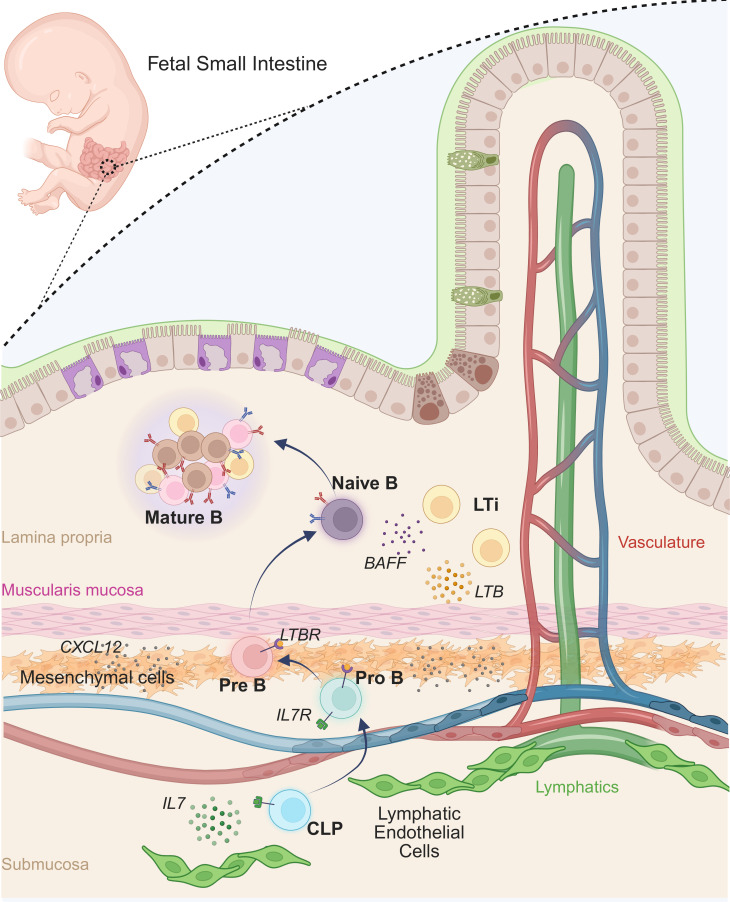

## Introduction

Postnatal development of mucosal tissues is now accepted as a critical window for shaping local immune responses and tissue homeostasis (1, 2). While it was originally assumed that fetuses possess a more primitive and restricted immune response during gestation than postnatal periods, recent studies have shown that this is not the case. Rather, a complex and unique immune system emerges during fetal development, containing cells from both innate and adaptive arms (3–5). Initial studies focused on describing the distribution of these cells in pre- and postnatal tissue, but their ontogenesis was presumed to be a result of hematopoiesis in the fetal liver. It was, thus, surprising when de novo B cell lymphopoiesis was discovered as a feature of embryonic small intestines (SI), a process that was once thought to exclusively occur postnatally, or in designated primary immune organs (1, 3, 6).

B cell lymphopoiesis is a dynamic process, that occurs continuously during the human lifespan, generating an essential arm of the immune system (7, 8). Canonical B cell lymphopoiesis has been extensively described in the bone marrow and, to a lesser extent, in the fetal liver, where common lymphoid progenitors (CLP), undergo cell differentiation in response to local molecular cues that drive B cell lineage commitment and proliferation (9, 10). In the bone marrow, the earliest B cell precursors, known as pre-pro–B cells, are guided by the chemokine CXCL12, produced by local stromal cells. This signaling prompts the upregulation of IL-7R, transitioning pre-pro–B cells into pro-B cells that are responsive to cytokines like IL-7 and Thymic Stromal Lymphopoietin (TSLP) (11). These cytokines are crucial for B cell lineage commitment, as they lead to the activation of the pre-B cell receptor (pre-BCR), ensuring that each developing B cell expresses only one functional heavy chain allele, an essential step in defining pre-B cell identity (12, 13). Once light chain rearrangement is complete, the cell is considered an immature B cell, which then migrates to the periphery and matures into a naive B cell through additional signaling events (14).

The mechanisms governing definitive B cell lymphopoiesis are largely conserved in humans and mice (15). Mutations in IL-7R, CXCR4, or CXCL12 in both mice and humans lead to severe immunodeficiencies and cancer due to impaired B cell development in the bone marrow and fetal liver (15, 16). However, the discovery of a complete B cell developmental trajectory, including receptor recombination via local RAG expression in neonatal intestines challenged this view and demonstrated that mucosal sites may be capable of noncanonical lymphopoiesis (6). More recently, the presence of developing B cell populations in human fetal intestines further suggests that the foundations of intestinal immunity are, in fact, locally and embryonically established. This is especially important considering the substantial role that early-life B cell–mediated responses play in maintaining intestinal homeostasis and protecting against pathogens (17, 18). Yet, whether this embryonic B cell lymphopoiesis program in the SI relies on canonical signaling and developmental cues remains unknown, and it is equally unclear whether these processes are conserved in model organisms.

Using single-cell RNA-Seq (scRNA-Seq), in situ immunofluorescence (IF), RNAscope, and high-dimensional spectral flow cytometry, we evaluated human and mouse intestinal tissue at fetal time points to investigate the composition and phenotype of B cells throughout gestation. We show that B cells develop in fetal mouse intestines in a manner akin to human tissue. Furthermore, we find that murine intestinal B cells exhibit phenotypic markers of stable tissue residency, distinguishing them from their counterparts in other hematopoietic sites, such as the fetal liver. Intriguingly, we demonstrate that lymphatic endothelial cells (LECs) express higher levels of *TSLP* and *IL-7* that are known to support local B cell proliferation, in addition to other factors that can facilitate the recruitment of CLPs to the tissue. Furthermore, we also show that fetal-derived lymphoid tissue inducer (LTi) cells are necessary for the maturation of B cell precursors in intestines. A model of LTi deficiency reveals a profound reduction in the B cell developmental program within the embryonic SI, thereby expanding the known roles of LTi cells at this site. Importantly, we confirm that LTi cells and their key cytokines that can regulate murine B cell development are also present in human fetal intestinal tissue, further underscoring the importance of this local process. This work identifies essential and conserved constraints to the establishment of an intestinal immune system and how B cell responses may be shaped far earlier in life than previously appreciated.

## Results

### CLPs seed human SIs late in the first trimester and develop into B cells.

**Figure 1:**
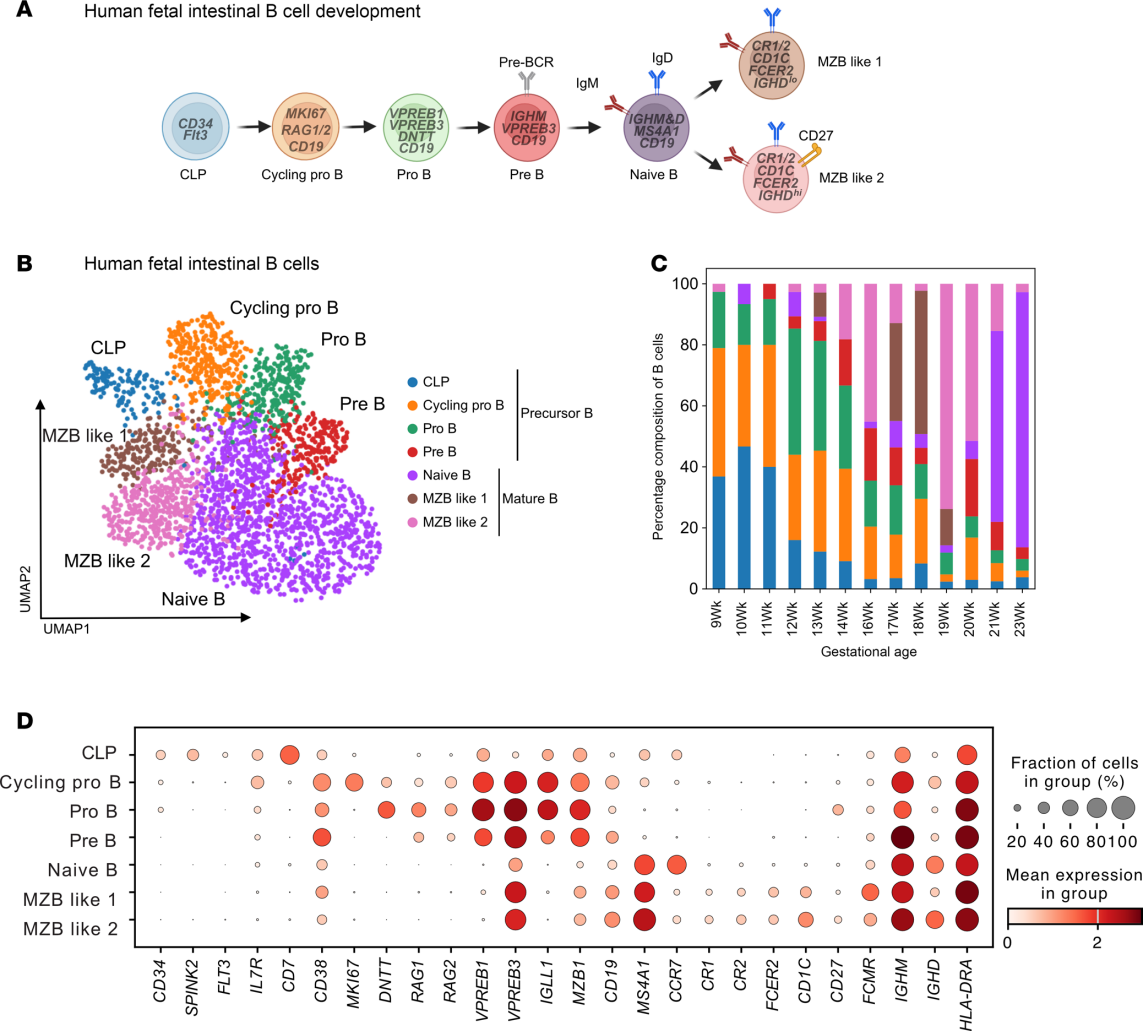
Characterization of fetal B cells in the SI. (**A**) Graphical diagram depicting canonical B cell developmental stages as it occurs in humans. (**B**) Uniform Manifold Approximation and Projection (UMAP) visualization of fetal B cells across all developmental time points. (**C**) Bar plot of relative proportions of B cell types at each developmental stage. (**D**) Dot plot of marker genes expression in fetal B cells. Color represents normalized mean expression of marker genes in each cell type, and size indicates the proportion of cells expressing marker genes.

We compiled a single-cell RNA-Seq (scRNA-Seq) atlas of the human SI across the human life span from 8 weeks estimated gestational age (EGA) to adulthood (19). Within this atlas, we identified B cells in the fetal tissue, albeit at lower proportions than in postnatal samples (3, 19). To comprehensively evaluate the presence of developing B cells in fetal intestinal tissues, we established strict immunological criteria to classify cells based on the expression of genes and proteins associated with B cell phenotypes and immunoglobulin gene recombination (Figure 1A) (14).

We found all components of the B cell developmental trajectory, including CLPs, characterized by the expression of precursor genes, *IL7R*, *CD7*, *CD34*, *SPINK2*, and *FLT3* (20–24); cycling pro- (*MKI67*^+^), pro-, pre-B cells; and mature B cells in the human fetal SI by scRNA-Seq (Figure 1, B–D). Pro-B cells were identifiable by their expression of *CD19* (a key B cell marker), along with surrogate light chain proteins such as *VPREB1*, *VPREB3*, and *IGLL1*, as well as the recombination enzymes *RAG1* and *RAG2* in concordance with prior studies (14, 25). Pre-B cells were identified, as previously defined (8, 25), by increased expression of surrogate light chain proteins and higher levels of *IGHM* indicating the ongoing assembly of the pre-B cell receptor (pre-BCR). Finally, mature B cells, including naive B cells and marginal zone B (MZB) like cells, lost expression of receptor recombination factors *RAG1/2* while increasing expression of *CD19*, *MS4A1* (encodes CD20), *IGHM*, and *IGHD* (14, 25). Naive B cells were characterized by the expression of *CCR7*, whereas MZB-like (MZB-like 1 and MZB-like 2) cells were identified by the expression of *CR1* (aka CD35), *CR2* (aka CD21), and *FCER2* (aka CD23), as well as *CD1C* (26). Notably, the MZB-like 2 cells exhibited higher expression of *IGHD*, suggesting a more mature or further developed status compared with MZB-like 1 cells. Altogether given the gestational age, these data support the likelihood that B cells are naive subsets, although we cannot exclude the possibility that they have memory features, not noted here. These data confirm that, in addition to the fetal liver and bone marrow, the fetal SI acts as a local niche for B cell development.

Interestingly, using temporal cell mapping analysis, PHATE, we show that the SI CLPs gave rise to cycling pro-B cells, plasmacytoid dendritic cells (pDCs), and innate lymphoid cells (ILCs) but not T cells (Supplemental Figure 1, A–C; supplemental material available online with this article; https://doi.org/10.1172/jci.insight.192550DS1). Furthermore, the precursor B cells (CLPs, cycling pro-B, pro-B, and pre-B cells) decreased while mature B cells (naive, MZB-like 1 or 2 B cells) increased across gestation, where at 9 weeks EGA, over 90% of B cells were of the precursor phenotype and by 23 weeks over 80% were of the naive phenotype (Figure 1C). Intestinal pro-B cells had higher expression of *DNTT*, *RAG1*, and *RAG2*, a set of genes that are critical for B cell development as they promote survival and proliferation of B cells and regulate immunoglobulin gene rearrangement (Figure 1D) (27).

### B cell development is a conserved feature present in murine fetal intestines.

**Figure 2:**
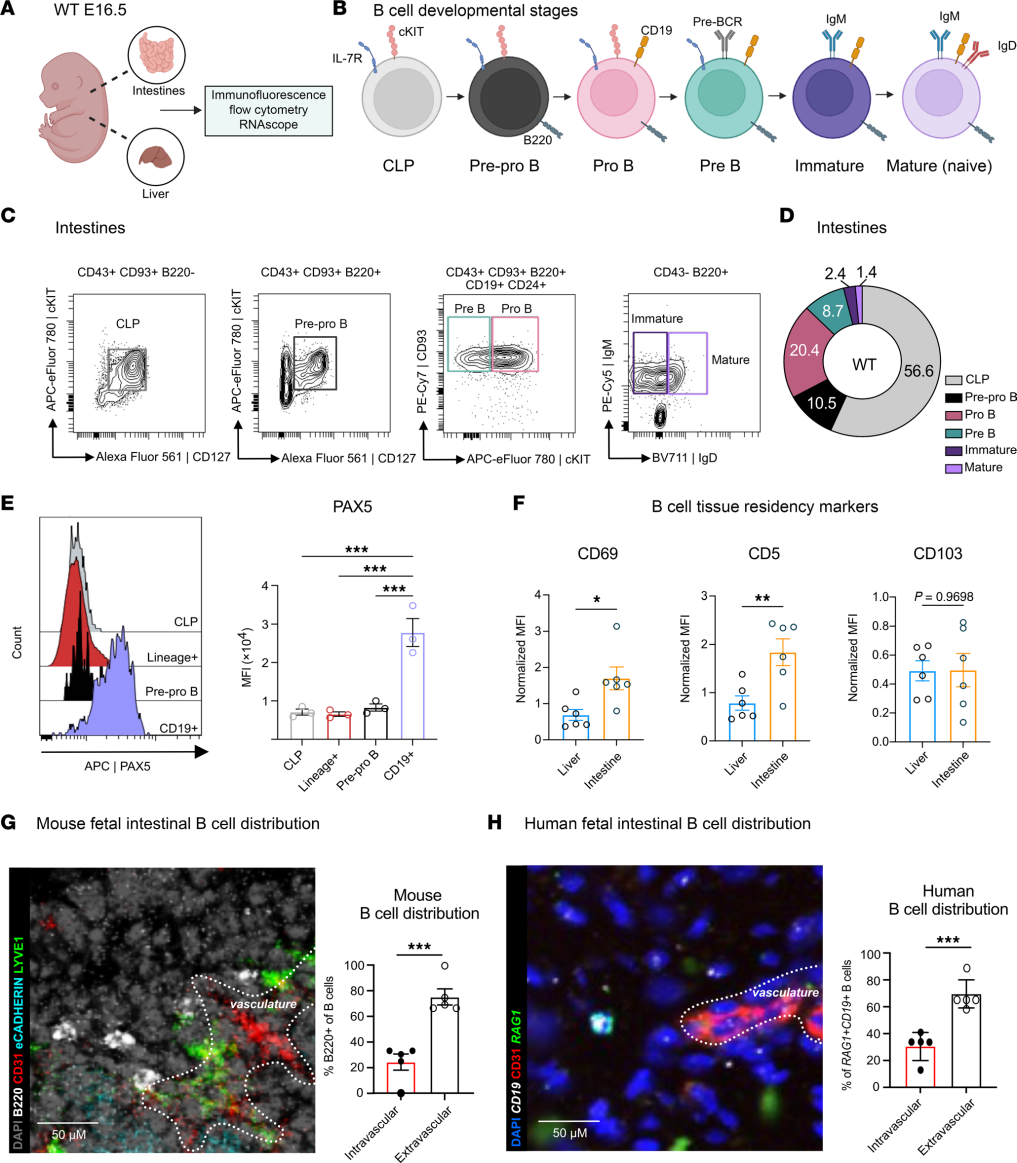
Fetal mouse intestines recapitulate human B cell development. (**A**) Schematic for tissue isolation and downstream analyses for mouse B cell analysis. (**B**) Graphical diagram depicting canonical B cell developmental stages as it occurs in mouse bone marrow. (**C**) Flow cytometry gates to identify distinct intestinal B cell subsets at E16.5; previous gating filters are listed above individual plots and depicted in Supplemental Figure 2A (*n* = 3, 6–8 pooled littermate tissues). Parent gates from live, CD45^+^lineage^–^ cells are listed above each plot. (**D**) Pie chart with average frequencies of total B cells from gates in Figure 2D (*n* = 3). Each color corresponds to a specific cell population, and numbers are listed as a percentage of total B cells. (**E**) Histogram overlay of PAX5 expression across CLP, lineage^+^, Pre-pro–B cells, and CD19^+^ B cells (B220^+^CD19^+^ B cells) with quantification of MFI, (*n* = 3, 1-way ANOVA, ***P < 0.001, data are shown as mean ± SEM). (**F**) MFI of CD69, CD103, and CD5 on E16.5 B cells (CD45^+^CD19^+^B220^+^ live cells) (*n* = 6 pooled litters, unpaired Student’s *t* test, *P < 0.05, **P < 0.01 data are shown as mean ± SEM). (**G**) Representative image from IF staining of fetal mouse intestinal tissue (E18.5). Full image in Supplemental Figure 4F. The white dotted outline marks vasculature. Pseudo-colored with anti-B220-FITC (white), anti–CD31-Alexa Fluor 555 (AF555) (red), anti–CD324-AF594 (cyan), and anti–LYVE1-AF647 (green) and counterstained with DAPI (gray) with quantification of intravascular (CD31^+^) versus extravascular (CD31^–^) B cells (*n* = 5, paired Student’s *t* test, ***P <0.001; data are shown as mean ± SEM (**H**) Representative image from fetal human intestinal tissue with IF staining CD31 (red) and probed with RAG1 (green) and CD19 (white); nuclei stained with DAPI (blue) to identify B cells. The white dotted outline marks vasculature. B cells were identified as intravascular, based on direct colocalization with CD31 (CD19^+^RAG1^+^CD31^+^), or extravascular, (CD19^+^RAG1^+^CD31^–^) and quantified (*n* = 5, paired Student’s *t* test, ***P <0.001; data are shown as mean ± SEM). Scale bar: 50 µM.

To understand if local lymphopoiesis at this time point is conserved in mammalian species, we next investigated whether intestinal B cell development can be observed in fetal mice as it is in humans. We isolated fetal livers and intestines from WT mice at E16.5, a time point at which definitive B cell development occurs primarily in the fetal liver (28). Tissues from fetal mice were analyzed by high-dimensional spectral flow cytometry (15–20 fluorophores), IF, and RNAscope, whereby developmental stages of B cells were demarcated by expression of key cell-surface markers (29) (Figure 2, A and B).

We first gated on CD45^+^ cells and selected lineage marker–negative cells to exclude non-B cells (Supplemental Figures 2 and 3). CLPs were distinguished from other immune populations by the lack of B220 (CD45R) and B-lineage markers and by positive expression of c-KIT and CD127 (IL-7R). To our knowledge, no recent studies have defined a comprehensive and specific flow cytometry–based gating strategy for B cell precursors in the fetal liver. Therefore, we adapted established markers commonly used in the bone marrow, spleen, and peritoneum (8, 30, 31), including B220, CD43, IgM, IgD, CD93, CD24, CD19, cKIT, and IL-7R (Supplemental Figure 3A). Early B cell stages (pre-pro–, pro-, and pre-) were distinguished from late stages (immature and mature) based on CD43 expression, with early subsets CD43^+^ and later subsets CD43^–^. Within the early CD43^+^ subsets, pro- and pre-B cells were identified as CD19^+^CD24^+^, while pre-pro–B cells are CD19^–^CD24^–^ but express c-Kit and CD127. Within these early subsets, cKIT expression was used to further separate pro-B (cKIT^hi^) from pre-B (cKIT^lo^) cells (32). Finally, levels of IgM and IgD expression defined immature and mature B cells (Figure 2C).

Using this strategy, we identified all major B cell subsets in both the fetal liver and intestine at E16.5. Notably, the fetal intestine exhibited the entire developmental range of B cells from CLPs to mature B cells at comparable numbers with some enrichment of CLPs (Figure 2D and Supplemental Figure 2, B and C). In contrast, the fetal liver was predominantly enriched for pro- and pre-B cells with diminishing percentages of other subsets (Supplemental Figures 3, A–C). We noted that while IgD was expressed in the mature B cells in the fetal intestines (Figure 2C), this level was lower than is usually observed in bone marrow (27). These findings highlight a spatially distinct developmental trajectory in the intestine, supporting its role as a de novo B cell developmental niche eventually resulting in the emergence of a local, naive B cell pool.

We next confirmed the identity of the B cell subsets above via expression of a lineage-specific transcription factor, PAX5, which represses non-B cell lineage genes while promoting the expression of B cell lineage genes (33). PAX5 is measurably expressed at the transition from pre-pro–B to pro-B stage (33). We found that intestinal B cells past the pre-pro–B cell stage (B220^+^CD19^+^) had significantly higher expression of PAX5 compared with CLPs, other lineage^+^ cells, and pre-pro–B cells (Figure 2E), therefore confirming their identification by expression of a B-lineage transcription factor in addition to surface proteins.

Given the essential role of early-life intestinal B cells in regulating local immunity and maintaining homeostasis through antibody- and receptor-mediated functions (17), we sought to characterize how developing intestinal B cells differ from those at other sites. Using immunophenotyping, we compared B cells from the fetal intestine and liver at E16.5. The fetal liver serves as a primary site for B-1a cell development, an innate-like B cell population found in serosal cavities, spleen, and various nonlymphoid tissues (34, 35). These cells are defined by their expression of CD5, CD43, and IgM, along with a germline-encoded polyreactive antibody repertoire (35). Spectral flow cytometry identified B-1a cells in both fetal liver and intestine (Supplemental Figure 2A and Supplemental Figure 3A). Intestinal B cells (B220^+^CD19^+^) exhibited higher CD5 mean fluorescence intensity (MFI) than their liver counterparts (Figure 2F and Supplemental Figure 4, A–C). Additionally, they showed significantly elevated CD69, a marker of tissue residency and early activation, while CD103, an epithelial adhesion protein, was not significantly increased, suggesting these cells are tissue resident but not strongly adherent to the intestinal epithelium (Figure 2F and Supplemental Figure 4, D and E).

### Murine intestinal fetal B cell development results in a predominately extravascular pool of heterogenous, resident B cells.

To determine where intestinal B cells reside in fetal mouse intestines and their proximity to intestinal stromal cells, we performed IF staining using cell-specific protein markers. Stromal cells were identified by their preferential expression of identifying markers: E-cadherin for intestinal epithelial cells (IECs), CD31 for vascular endothelial cells, LYVE1 for lymphatic endothelial cells (LECs), and B220 for B cells. Most B cells were found within the submucosal layer of the intestines (Figure 2G and Supplemental Figure 4F). Within this layer, B cells were observed to be predominantly perivascular and showed mixed distribution throughout the tissue (Figure 2G and Supplemental Figure 4F). Notably, fewer B cells directly overlapped with CD31^+^ cells, suggesting that they are likely tissue-residing rather than circulating within the intestinal vasculature. Within the human intestines, a similar phenomenon was observed with the majority of *CD19^+^RAG1^+^* cells residing in extravascular areas (Figure 2H and Supplemental Figure 4G). These data reveal that mouse intestines, like human intestines, provide an additional site for B cell development during embryogenesis, where developing and mature B cells reside in the tissue as extravascular foci. Furthermore, the B cells generated in this tissue have unique phenotypic properties and are a mix of B cell subsets, likely indicative of their distinct contributions to tissue specific roles, further distinguishing them from their fetal liver counterparts.

### Local stromal cells are capable of supporting B cell development in the fetal SI.

**Figure 3:**
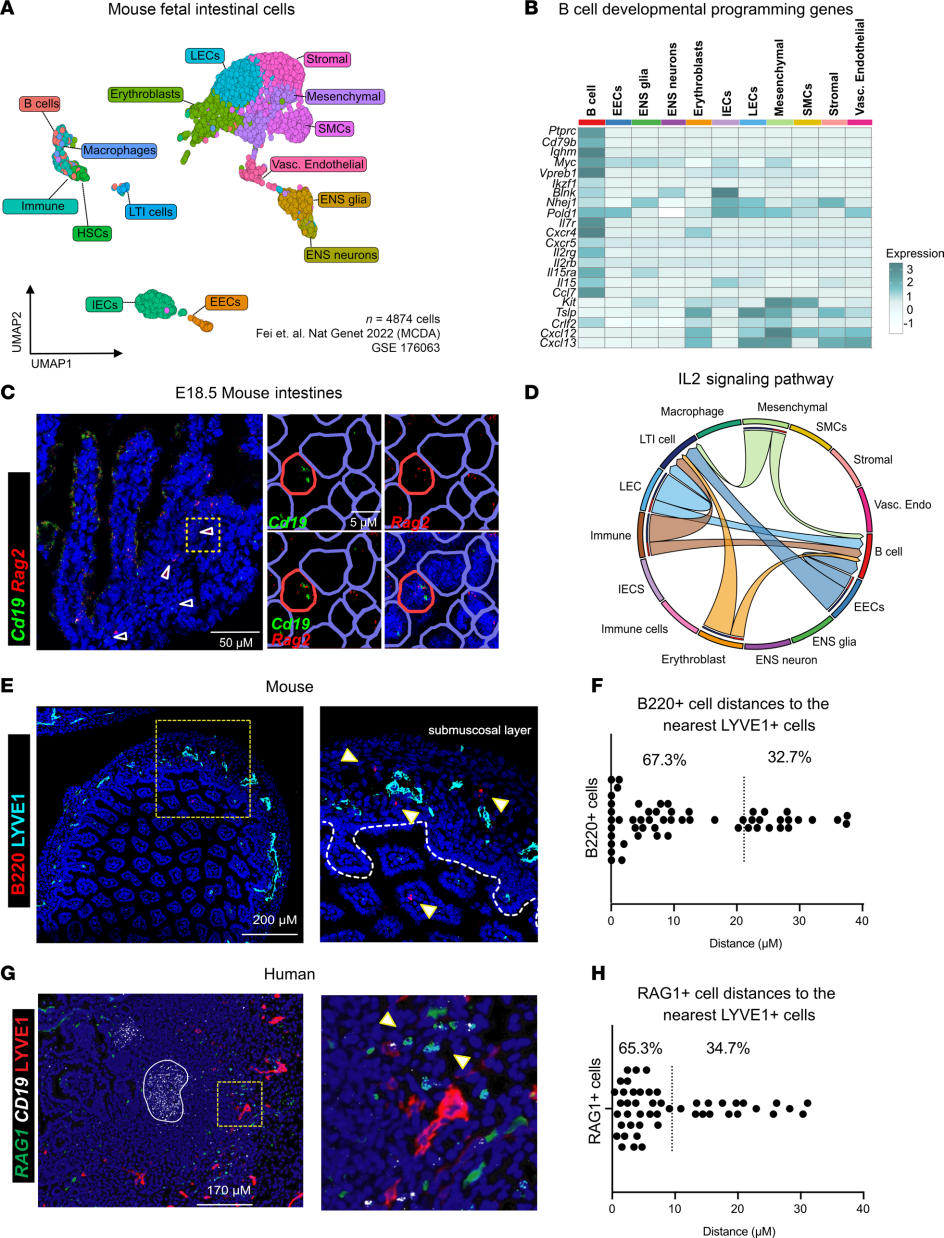
Epithelial and stromal cells express molecular signals that may support B cell development in fetal intestines. (**A**) UMAP of scRNA-Seq data from publicly available MCDA E14.5 fetal intestines with cell populations identified by differential gene expression (Supplemental Figure 4). (**B**) Heatmap of differential gene expression between B cells and subsetted stromal cells using curated gene list of different receptor ligand pairs involved in bone marrow B cell development. (**C**) Representative multiplex RNAscope images of fetal mouse intestine (E18.5). The yellow square highlights the region magnified on the right. Blue circles denote cells, red circles denote *Cd19^+^* and *Rag2^+^* cell. Scale bar: 50 μm (left) or 5 μM (right). (**D**) Chord diagram depicting the predicted IL-2 family signaling interactions between sender and receiver populations with colors corresponding to the sender population (outer ring) and arrowheads pointing to the receivers based on total cells and highlighted receptor ligand pairs within the predicted interactions. (**E**) Immunofluorescence of representative mouse intestinal tissue from E18.5 mice showing nuclear stain DAPI (blue), B220-FITC (red), and LYVE1-AF555 (cyan) with a selected, zoomed ROI with arrowheads to identify B cells. (**F**) Measured distance to nearest LYVE1 LECs with dots corresponding to individual cell distances (*n* = 3) with mean distance highlighted by dotted line and reported percentages below and above the mean. (**G**) Human intestinal tissue (21 weeks gestational age) stained with RNA probes RAG1 (green), CD19 (white), and anti-LYVE1 antibody (red). (**H**) Nuclei counterstained with DAPI (blue) in an identical analysis to **F** in human tissue sections (*n* = 1). Scale bars: 50 µM (**C**), 200 µM (**E**), 170 µM (**G**).

Having confirmed the intestines as an additional site for B cell development, we next questioned what cellular or molecular mechanisms exist in this distinct location to facilitate lymphopoiesis. Fetal liver and bone marrow B cell lymphopoiesis rely on cues from local stromal cell populations to promote B cell survival, migration, and maturation (11, 24, 27). We leveraged published scRNA-Seq datasets of total intestinal tissue at E14.5 from Mouse Cell Developmental Atlas (MCDA) for an unbiased approach to predict interactions between developing B cells and other intestinal cell types (36). A Uniform Manifold Approximation And Projection (UMAP) was used to view the high-dimensional data, and distinct populations were classified based on differential gene expression of characteristic transcriptional markers as well as top differentially expressed genes (Figure 3A and Supplemental Figure 5, A and B). We identified B cells by expression of *Ighm* and *Cd79b*, and we further specified macrophages by expression of *Adgre1* (F4/80). Cells that expressed immune-related genes, but not lineage-specific markers, were broadly classified as “Immune” and cells expressing *Cd34* were defined as hematopoietic stem cells (HSCs) (Supplemental Figure 5A). In addition to these immune cells, we identified LTi cells, by *Rorc* expression. We also identified a variety of epithelial and stromal populations in this dataset, such as IECs, enteroendocrine cells (EECs), enteric nervous system (ENS) cells, LECs, and smooth muscle cells (SMCs) (Supplemental Figure 5A).

To determine how intestinal B cells may interact with stromal cells at fetal time points, we evaluated interactions between of B cells and stromal cells in the intestinal scRNA-Seq dataset. We then queried the expression of genes encoding receptor and ligand pairs that are required for bone marrow B cell–lymphopoiesis (27). We noted that fetal intestinal B cells expressed *Ikzf1* (gene encoding IKAROS), a key transcription factor required for pre-B cell differentiation (37). We also identified the expression of *Il7r* in B cells, a receptor required for B cell development, survival, and proliferation (Figure 3B) and confirmed its expression in fetal intestinal B cells in mice via spectral flow cytometry (Supplemental Figure 2A). Furthermore, intestinal B cells expressed genes associated with receptor recombination, including *Vpreb1*, *Blnk*, *Nhej1*, and *Pold1*, supporting the conclusion that embryonic intestinal B cells may be actively undergoing receptor recombination at this stage (Figure 3B).

Previous studies of B cell development in neonatal intestines have shown that local expression of the endonucleases RAG1 and RAG2 contributes to shaping the preimmune repertoire in response to microbial colonization (6). To investigate whether receptor recombination can occur independently of microbial exposure, we examined the expression of *Rag2* in B cells residing in fetal intestinal tissue by spatial transcriptomics. Using RNAscope, we identified B cells in fetal intestines by *Cd19* expression (Figure 3C). Notably, approximately 25% of these B cells expressed *Rag2* in situ (Supplemental Figure 5C), indicating active recombination. We noted that these *Rag2*-expressing B cells primarily reside in the submucosa, akin to the distribution of B220 in these regions (Figure 2H and Figure 3E). These findings suggest that B cell receptor recombination may occur prenatally in mice, revealing a previously unrecognized site for the generation of the preimmune repertoire in the absence of microbial signals.

Having identified *Rag2*-expressing B cells, we attempted to identify the local source of key cytokines and chemokines that may facilitate their local maturation. Transcriptional levels of *Il7r* ligand, *Il7*, were not detectable in the fetal intestine dataset. However, we found that *Tslp* (encodes TSLP), the primary IL-7R ligand in the fetal liver (38), was expressed by a variety of cells in mouse intestines and particularly by LECs (Figure 3B). We used the computational package, CellChat to predict the relative contribution of cells that were sending or receiving signaling through the *Il7r/Tslp* (IL2 signaling family) axis (Supplemental Figure 5, D–F). In these circos plots, the orientation of the arrows denotes the direction of the signaling of ligands to their cognate receptor, expressed by other cell types. This analysis reveals that B cells are likely receiving IL2 family signals predominantly from LECs and an unspecified immune cell subset (Figure 3D and Supplemental Figure 5F) (39). Further examination of the enriched genes in this signaling node reveals that *Tslp* expressed by LECs is predicted to signal to intestinal B cells through *Il7r* and *Crlf2*, which form a heterodimeric receptor for TSLP (12) (Supplemental Figure 5F). This reveals a putative signaling node, specific to the intestines that could facilitate embryonic intestinal B cell proliferation.

As proximity of B cells and LECs is predicted during postnatal Peyer’s patch (PP) or gut-associated lymphatic tissue (GALT) development in murine intestines (40), we leveraged IF staining in fetal mouse tissue to determine if B cells specifically colocalize with LECs during early embryonic developmental windows. We noted that B cells (B220^+^) primarily resident in the submucosal layer form a close association with LECs (LYVE1^+^) in mouse intestines (Figure 3, E and F). The correlation of *Rag2*-expressing B cells in the submucosa, proximal to LECs, suggests a possibility that local prelymphatic structures may help support and organize B cell maturation or receptor recombination.

We then used RNAscope combined with IF to delineate where B cell maturation is taking place within the human SI. Consistent with the mouse data, we found that the precursor B cells (*RAG1*^+^*CD19*^lo^) predominantly localized in the submucosal layer, with over 65% being proximal to the endothelial cells (LYVE1^+^), while mature B cells (*RAG1^–^CD19^+^*) were aggregated proximal to the epithelial cells and distal to the endothelial cells (Figure 3, G and H). Intriguingly, while populations of precursor B cells in the submucosal layer are somewhat different between mice and humans, stark similarities in the localization profile of these precursor B cells between species suggest that the cellular and molecular cues that facilitate local *de novo* B cell development are evolutionarily conserved (Figure 3, E–H).

### LTi cells reside proximal to B cells in fetal intestines and are predicted to signal via chemotactic and lymphoid tissue promoting pathways.

**Figure 4:**
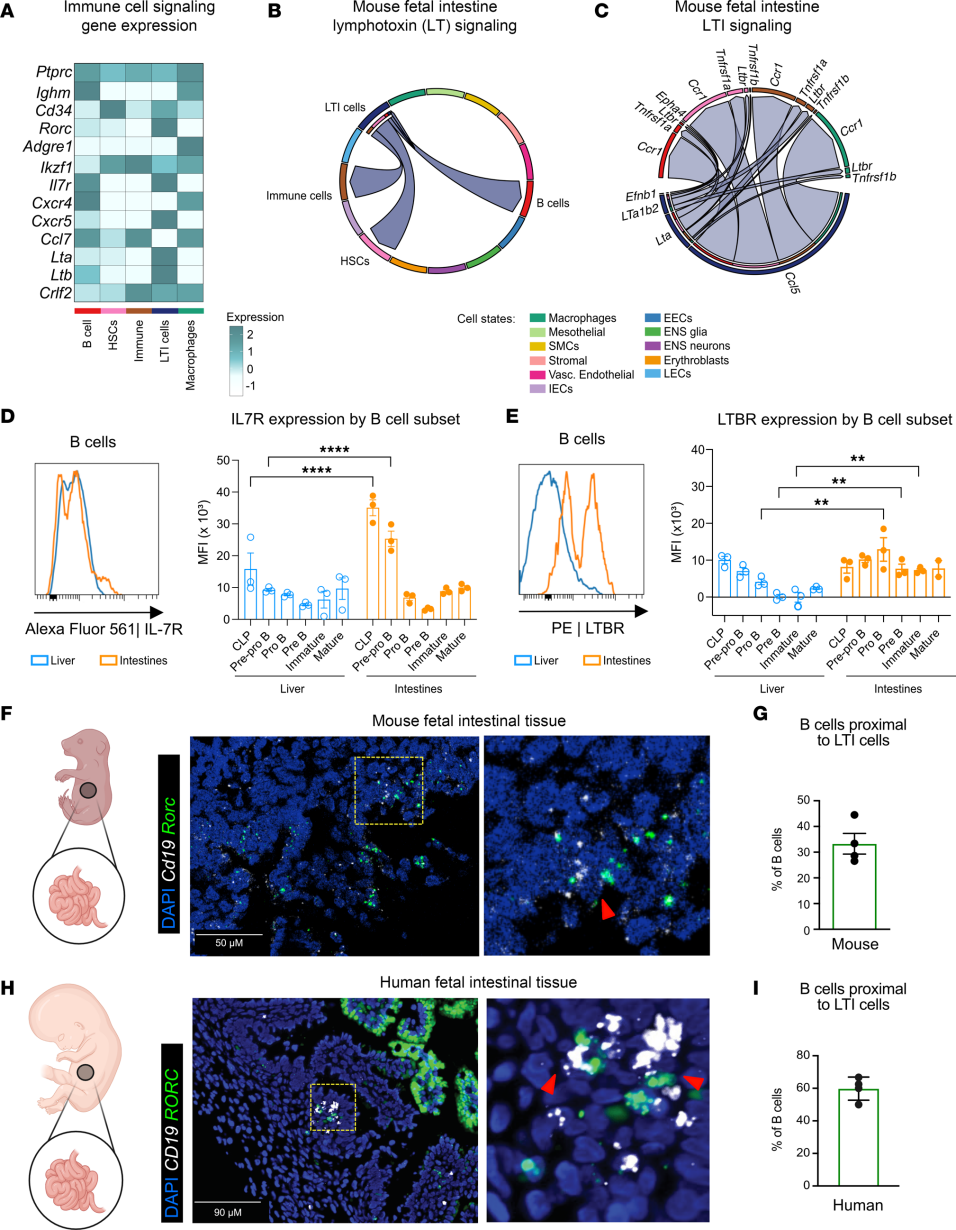
LTi cells in mouse intestines reside proximal to B cells with predicted interactions influencing B cell niche establishment at fetal time points. (**A**) A heatmap depicting the relative expression of queried receptor-ligand pairs from subset immune cell populations from mouse scRNA-Seq (Figure 3A and Supplemental Figure 4) (**B**) Chord diagram depicting the predicted lymphotoxin (LT) signaling interactions between sender (LTi cell) and receiver populations of LT family ligand and receptors from MCDA dataset with colors corresponding to the sender population (arrow direction corresponds to target populations, and flat ends correspond to sender population) and pointing to the receivers (outer ring color). (**C**) Chord diagram from CellChat of all ligand and receptor interactions from LTi cells to other intestinal cell populations (denoted by outer ring color). Arrowhead direction corresponds to predicted receiver. (**D** and **E**) IL-7R or LTBR expression in intestinal and fetal liver B cells (CD19^+^B220^+^ live cells) depicted in histogram and quantified MFI across B cell subsets (Figure 2C) (*n* = 3, 1-way ANOVA, **P < 0.01, ****P < 0.0001; data are shown as mean ± SEM). (**F**) Representative image from fetal mouse intestinal tissue (E18.5) mice stained with RNA probes *Cd19* (white) to mark B cells and *Rorc* (green) to mark LTi cells, counterstained with DAPI (blue). Yellow box outlines selected ROI depicted to the right. Scale bar: 50 µM. Red arrows denote LTi and B cell interactions. (**G**) Quantification of B cell and LTi proximity analyzed by determining the percentage of *Cd19*^+^ cells within 20 μM of *Rorc*^+^ cells divided by total *Cd19*^+^ cells. (**H**) Representative human intestinal tissue section (21 weeks gestational age) stained with RNA probes *CD19* (white) and *RORC* (green), counterstained with DAPI (blue) and a zoomed section outlined in yellow box, depicting LTi and B cell interactions in intestinal tissue. Scale bar: 90 µM. (**I**) Quantification of B cell and LTi proximity analyzed by determining the percentage of *CD19*^+^ cells within 20 μM of *RORC*^+^ cells divided by total *CD19*^+^ cells.

Given the close apposition of intestinal B cells and LECs during embryonic development, we next wondered if interactions between B cells and local cells were supported by a tissue scaffold within the mouse fetal intestines. Intriguingly, among the different populations that expressed higher levels of developmental drivers for B cell lymphopoiesis, LTi cells strongly expressed multiple ligands for B cells (Figure 4A). LTi cells belong to a family of ILCs (ILC3) that support lymphoid tissue scaffold development throughout the body but also specifically coordinate the formation of intestinal lymphoid follicles and PPs (41). Predictive analysis of these interactions revealed that lymphotoxin (LT) signaling was specifically enriched between B cells and LTi cells, with LTi cells as the preferential senders of LT ligands to receptors expressed by B cells (Figure 4B).

Development of lymphoid tissue often requires the expression of LTA1B2 by LTi cells to signal through LTBR on stromal organizer cells to promote cell aggregation and niche survival (42). However, in the bone marrow, LT signaling through membrane-bound LT heterotrimers (LTA1B2) and soluble LT-α homotrimers (LTA3) is shown to control B cell maturation and class-switching in adult mice (43, 44). Thus, we evaluated the various interactions that were upregulated between these populations. Notably, LTA (*Lta*) produced by LTi cells was predicted to primarily signal through TNF family receptors on B cells (Figure 4C). Furthermore, we found that LTi-derived *Lta* was predicted to signal through B cell–expressed *Ltbr*. Expression of LTA receptor, LTBR (*Ltbr*), on B cells can regulate their differentiation, specifically during the formation of plasma cells (45). Additionally, in recent human studies, biallelic loss-of-function mutations in LTBR were found to be associated with decreased B cell differentiation (46).

Building on our identification of IL-7R as a receptor involved in B cell proliferation, we investigated whether intestinal B cells express LTBR and whether the expression of IL-7R and LTBR varies across different stages of B cell development. As expected, we found that expression of IL-7R was more consistent across B cell populations, including pro-B, pre-B, immature, and mature B cells between intestines and livers (Figure 4D and Supplemental Figures 2 and 3). However, the expression of LTBR was categorically higher in fetal intestinal B cell subsets compared with fetal liver counterparts, specifically starting at the pro-B cell stage (Figure 4E). This positions LTBR signaling as a putative tissue-specific receptor influencing B cell development at embryonic stages.

Considering that predictive analysis revealed that LTi cells were preferential senders of ligands for LTBR, we utilized RNAscope to expanded upon these data to see if we could visualize LTi and B cell interactions in situ. We detected that B cells (*Cd19*^+^) resided near LTi cells (*Rorc*^+^) in mice and human tissue (Figure 4, F–I), revealing that these cells are situated proximal to one another, likely capable of interacting through LTBR and LT-α. In mice, approximately one-third of B cells were found to colocalize with LTi cells, residing within 20 μm of each other and appeared to be positioned more closely to the intestinal epithelium than to the submucosa (Figure 4, F and G). In humans, approximately 60% of B cells were found near LTi cells (Figure 4, H and I). This stronger association may be attributed to species-specific developmental differences, as PPs form prenatally in humans, whereas in mice, they develop after birth (42).

When considered alongside the earlier observation that B cells are frequently associated with LECs, these data reveal a heterogeneous spatial distribution of developing B cells in situ. Specifically, early-stage B cells (~67%) tend to localize proximal to LECs in mice (Figure 3F), while a separate, likely more mature subset, shows stronger spatial associations with LTi cells subset (Figure 4, G and I). Altogether, these findings suggest that the fetal intestinal environment contains multiple microanatomical niches that support distinct stages of B cell development. The dynamic interactions between B cells and diverse stromal or immune cell types, such as LECs and LTi cells, highlight a complex and temporally regulated network of cellular cues that have the capacity to shape early B cell maturation and positioning prior to birth.

### LTi cells are required for complete B cell development in fetal mouse intestines.

**Figure 5:**
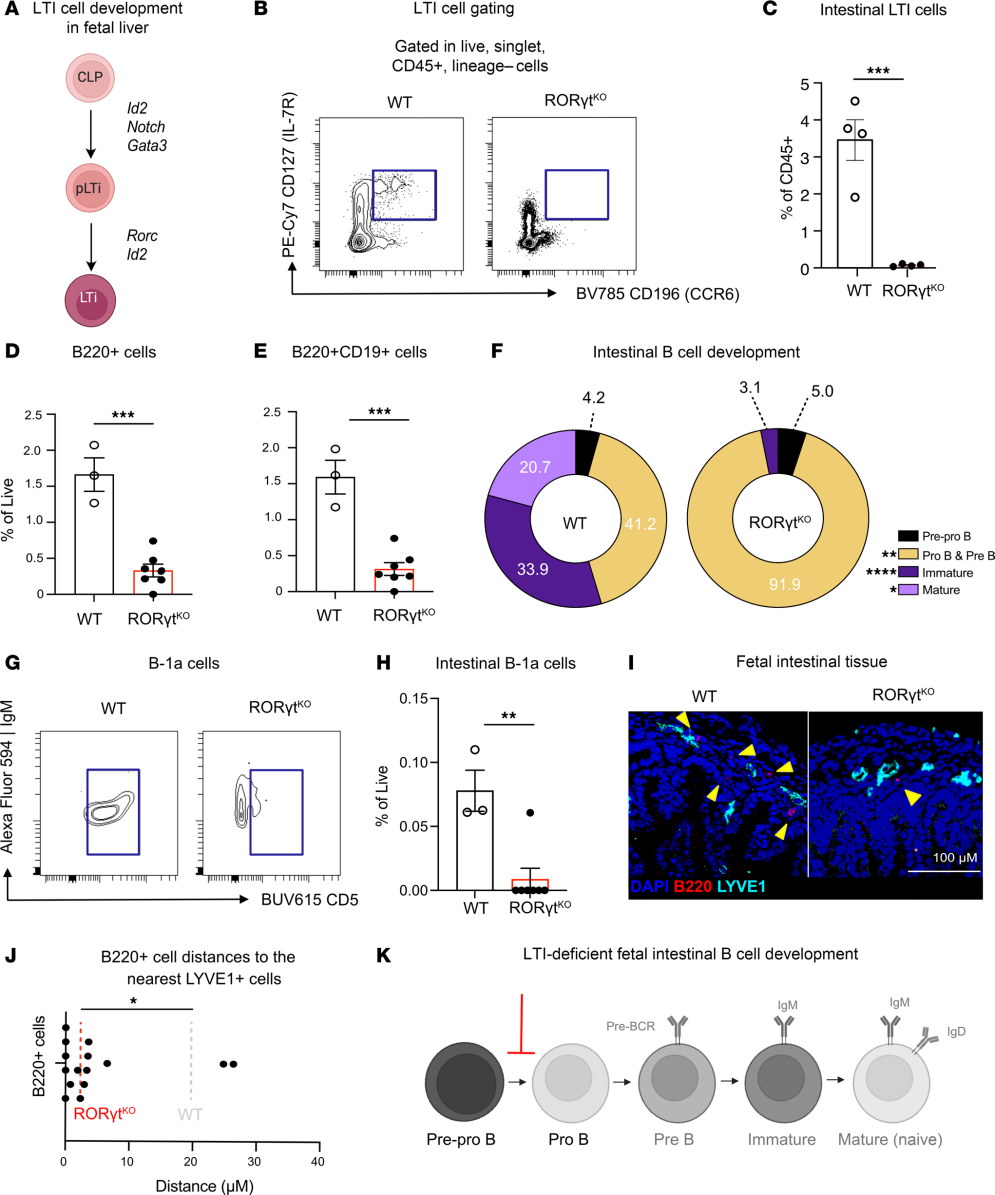
LTi cells are essential for intestinal B cell development in fetal mice. (**A**) Graphical diagram of LTi development as it is known in the fetal liver through transcriptional activation of RORγt. (**B**) Gating strategy to identify LTi cells in intestinal tissues of WT or RORγt^KO^ mice. (**C**) Percentage of LTi cells out of CD45^+^ cells based on gating in **B** by Student’s *t* test; ***P < 0.001 (*n* = 3–4). (**D** and **E**) Percentage of total B220^+^ cells (**D**) or B220^+^CD19^+^ (**E**) cells out of live cells based on gating in Supplemental Figure 2B. (Student’s *t* test, **P < 0.01, *n* = 3–7). (**F**) Pie chart with average frequencies of total B cells from gates in Figure 2D (*n* = 3–7) in WT or KO mice. Each color corresponds to a specific cell population, and numbers are listed as a percentage of total B cells. (**G**) Gating strategy to identify B-1a cells (CD45^+^, CD19^+^, and CD5^+^) in intestinal tissues of WT or RORγt^KO^ mice. (**H**) Percentage of B-1a cells out of CD19^+^ cells (Student’s *t* test, ****P < 0.0001, *n* = 3–4). (**I**) Representative immunofluorescence image of intestinal tissue (E18.5) from RORγt^KO^ mice showing nuclear stain DAPI (blue), B220-FITC (red), and LYVE1-AF555 (cyan) with a selected, zoomed ROI with arrowheads to identify B cells. (**J**) Measured distance to nearest LYVE1 LECs with dots corresponding to individual cell distances (*n* = 3) with mean distance highlighted by dotted line. Statistics calculated based on the average distance between B cells and LYVE1 cells within (*n* = 3–4) tissue sections (Supplemental Figure 6B), with Student’s *t* test; *P < 0.05. (**K**) Modified graphical diagram (Figure 2B) depicting reduction in B cell development in intestinal tissue. Scale bar: 100 µM.

Based on these findings, we next sought to test if LTi function in the embryonic intestines is in line with their known roles in B cell maturation and lymphoid tissue formation. Fetal LTi cells specifically require the transcription factor, RORγt to fully mature from hematopoietic precursors (41). Thus, we utilized RORγt-deficient mice (RORγt^KO^) that specifically lack LTi cells in the fetal windows due to a blockade in differentiation from fetal liver precursors (47) (Figure 5, A–C, and Supplemental Figure 7, A and B). Using flow cytometry of pooled embryonic intestinal tissues, we observe a significant decrease in intestinal B cells in RORγt^KO^ fetal mice when compared with WT (Figure 5, D and E, and Supplemental Figure 7, C and D). Evaluation of the relative composition of B cell subsets revealed that there was a reduction in early B cell differentiation, specifically after the pre-pro–B cell stage, in the RORγt^KO^ mice compared with WT controls (Figure 5F and Supplemental Figure 7D). Notably, this reduction was unique to the intestine, as B cell development in the fetal liver remained unaffected since no significant differences in B cell subsets were observed between WT and RORγt^KO^ mice in that tissue (Supplemental Figure 7, A and B). These findings underscore the critical, intestinal tissue–specific role of LTi cells in supporting B cell differentiation during fetal development. Furthermore, we saw that, of the remaining CD19^+^ intestinal B cells, there were significantly fewer B-1a cells (CD43^+^IgM^+^CD5^+^ B cells), indicating that this essential tissue resident B cell population, which is substantially reduced at E16.5 in RORγt^KO^ mice (Figure 5, G and H), is uniquely reliant on LTi. These data reveal that LTi cells are essential for intestinal B cell lymphopoiesis, distinct from the cells regulating bone marrow and fetal liver hematopoiesis.

We then queried whether B cell distribution is altered in RORγt^KO^ intestines. Upon analysis of the distribution of fetal RORγt^KO^ B cells in situ, we saw that B cells primarily localized to LECs (LYVE1^+^) (Figure 5, I and J, and Supplemental Figure 7C), significantly closer than the mean distance of intestinal B cells in WT counterparts (Figure 3, E and F). Thus, the absence of LTi cells in intestinal tissue results in an altered distribution pattern within the tissue, indicating altered migration in the tissue. Importantly, this suggests that LTi cells are involved in the development and patterning of B cells in intestinal tissue during embryogenesis, as shown by the reduced differentiation of precursor B cells (Figure 5K) and their increased association of B cells with LECs in RORγt^KO^ animals. Taken together, this identifies a tissue-specific cell type regulating B cell lymphopoiesis at this site.

### Fetal human intestinal cells can promote intestinal B cell development through mechanisms similar to those in mouse tissue.

Altogether, our data suggest several mechanisms and cell types that support B cell development in fetal mouse intestinal tissue. Thus, we next aimed to translate these findings to human intestines. We performed cellular-interaction analysis and noted that, in human SI, precursor B cells were more likely to interact with stromal cells and mature B cells were more likely to interact with LTi (NCR^+^ ILC3) cells compared with other immune cells (Supplemental Figure 8). We then used predicted receptor/ligand interactions from human scRNA-Seq data and we used RNAscope combined with IF to identify the various factors required for B cell lymphopoiesis.

**Figure 6:**
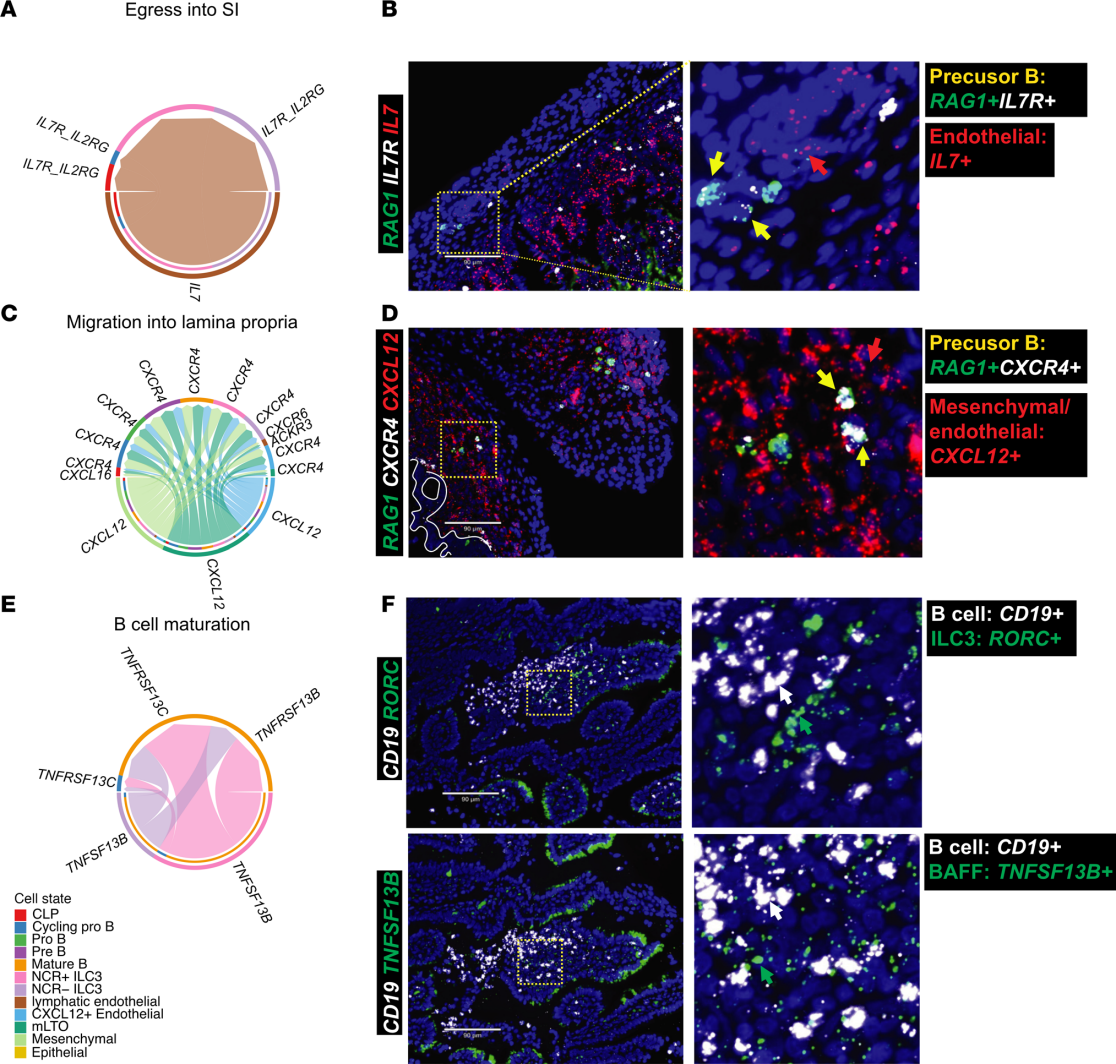
Humans share a common scaffold with mice that are capable of supporting B cell development in fetal intestines. (**A**, **C**, and **E**) Circle plots showing the selected interactions of IL-7 signaling (**A**), CXCL12 signaling (**C**), and BAFF signaling (**E**). (**B**) Representative multiplex RNAscope images of fetal small intestine (21 weeks gestational age). The yellow square highlights the region magnified on the right. Yellow arrows represent precursor B cells (RAG1^+^IL-7R^+^), red arrows yellow arrows represent IL-7^+^ cells. Scale bar: 90 μm. (**D**) Representative multiplex RNAscope images of fetal small intestine (21 weeks gestational age). The yellow square highlights the region magnified on the right. Yellow arrows represent precursor B cells (RAG1^+^CXCR4^+^), red arrows yellow arrows represent CXCL12^+^ cells. The white circle highlights the epithelial layer. Scale bar: 90 μm. (**F**) Representative multiplex RNAscope images of fetal small intestine (21 weeks gestational age). The yellow square highlights the region magnified on the right. All images counterstained with DAPI (blue). White arrows represent CD19^+^ B cells, green arrows in the upper image represent RORC^+^ cells, and green arrows in the lower image represent TNFSF13B^+^ cells. Scale bar: 90 μm.

We observed that *IL7-IL7R* interactions preferentially occurred between endothelial cells (*IL7^+^*) and CLPs (*IL7R^+^CD34^+^*) (Supplemental Figure 8, A–C, and Supplemental Figure 9, A–C), as well as cycling pro B cells (*IL7R^+^RAG1*^+^) in the submucosal layer (Figure 6, A and B), similar to the mouse intestinal tissue. This node may support the egress of CLPs into the SI. Within the intestine, we also found that *CXCR4* was expressed by all the different subsets of B cells, and its ligand *CXCL12* was expressed by some mesenchymal and endothelial cells (Figure 6C) with expression broadly distributed across the submucosal layer (Figure 6D). This corroborated what we saw in the mouse scRNA-Seq, where B cells highly expressed *Cxcr4*, a chemokine receptor for B cell migration, and stromal populations had higher transcription of the chemokine *Cxcl12*, the cognate receptor, indicative of their participation in B cell recruitment to the tissue (Figure 3B). We identified precursor B cells (*CXCR4^+^CD34^+^* and *CXCR4^+^RAG1^+^*) proximal to the submucosal layer, while more mature cells marked by *CXCR4^+^RAG1^–^* were proximal to the epithelial layer (Figure 6D and Supplemental Figure 9D), suggesting that this interaction may support the migration of B cells as they mature from the submucosal to the mucosal layer. This also highlights a difference between human and mouse biology at fetal time points, where larger B cell clusters are found in the lamina propria of the former and undetectable in the latter.

Finally, to determine what signaling is leading to mature B cell retention in the lamina propria, we examined B cell activating factor (BAFF) signaling in the intestines as it is critical for B cell survival and homeostasis (48). BAFF (encoded by *TNFSF13B*) was predominantly expressed in LTI (NCR^+^ ILC3) cells, and its receptor, BAFFR (encoded by *TNFRSF13C*) was enriched for in mature B cells (Figure 6, E and F, and Supplemental Figure 9B), an interaction that is previously described in spleen (49) but not in intestinal tissue. Using RNAscope, we identified that B cells (CD19^+^) present in the lamina propria were adjacent to BAFF producing ILC3s (*RORC*^+^; *TNFSF13B*^+^), another immune interaction in this tissue at this embryonic time point (Figure 3D). Collectively, our data suggest that the SI acts as a local hematopoietic niche for B cell development from CLPs to mature B cells supported by IL-7, CXCR4, and BAFF signaling, utilizing mechanisms that are shared with hematopoiesis occurring in the bone marrow and conserved between fetal mouse and human intestinal tissue. 

## Discussion

Tissue-autonomous immunity plays a crucial role in maintaining organismal health, especially at barrier sites such as the intestines, lungs, and skin (50–52). Resident immune cells at these sites act as first responders to pathogens and danger signals, but they are also equipped with various mechanisms to maintain homeostasis by resolving inflammation and tolerating commensal microbes (52). Recent studies have shown that, at birth, the intestinal B cell response is both diverse and essential for the early establishment of the microbiome (18). In fact, local B cell development is a described feature in neonatal intestines where microbialization influences the repertoire and function of early life B cells (6). Moreover, these cells primarily perform homeostatic functions and support the development of early immune memory—functions that cannot be fully replaced by bone marrow–derived B cells (17). Together, these findings underscore the importance of early life in shaping immune responses, yet much remains unknown about how these responses are established prenatally, in a microbial-independent manner.

Our study uncovers the fetal intestine as a previously unrecognized site of B cell lymphopoiesis, influenced by tissue-specific mechanisms. While previous studies have documented the presence of B cells at various stages of development in different tissues (1, 5, 19), the factors that govern this process have remained poorly understood. In this work, we demonstrate that B cells in the fetal intestine develop in response to the same cytokines and chemokines as those in the bone marrow. Notably, however, the fetal intestinal LECs and mesenchymal cells emerge as the putative sources of these early signals for B cell proliferation and migration. Furthermore, we show that LTi cells, an immune cell subset essential for the early formation of secondary lymphoid organs, are critical for regulating the complete development of intestinal B cells, likely through LT and chemokine signaling. This contrasts with bone marrow B cell development, where stromal cells provide similar signals to maintain the bone marrow microenvironment, supporting full B cell maturation (53). While these signaling pathways likely influence B cell differentiation directly, as indicated by the expression of receptor-ligand pairs, it is plausible that LT production also has indirect effects on the intestinal environment that contribute to the phenotype observed in RORγt-deficient embryos. These unique features of SI B cell lymphopoiesis further underscore our designation of developing B cells as immature/mature, though based on classical marker distribution, they may in fact represent distinct stages of development in B cell populations from those defined in traditional hematopoietic niches. These hypotheses remain to be explored in future studies.

Furthermore, our study reveals that intestinal B cell differentiation occurs in a nearly identical manner in both fetal humans and mice, underscoring the importance of this conserved mechanism. In our integrated human analysis, we observe that intestinal CLPs specifically develop into early B cell populations in situ. This finding aligns with recent studies suggesting that different subtypes of CLPs preferentially give rise to distinct lymphoid populations (54). Specifically, developmentally restricted HSCs (drHSCs) are known to favor the generation of innate-like B and T cells, which are critical for immunoregulatory functions, particularly in self-recognition and tolerance (54). Based on our results, we propose that these intestinal CLPs, as drHSCs, acquire their functional identity through tissue-intrinsic developmental processes that enable them to respond effectively to local antigens. We hypothesize that disruptions to this program could lead to immune dysregulation, manifesting as autoimmunity, dysbiosis, or allergy, because of abnormal B cell phenotypes and antibody specificities. This interpretation is supported by studies showing that RORγt-deficient animals (47) and LTBR-deficient mice (46) exhibit impaired B cell responses and increased susceptibility to infections. Our observations show that B cell seeding into the tissue occurs in a coordinated manner prior to microbial colonization. In this regard, we propose that the location that the prenatal intestinal B cells reside in may mark future sites for lymphoid aggregates or PPs, providing a necessary niche for the organization of mucosal immune responses.

Importantly, we observed several distinguishing features in intestinal B cell development between mice and humans, which may partially stem from species-specific differences in secondary lymphoid tissue formation during prenatal development (42). Notably, we found a significant increase in the colocalization of B cells with LTi cells in human fetal tissue compared with that of fetal mice. Interestingly, the proportion of B cells in contact with LTi cells closely mirrored the relative distribution of immature and mature B cell subsets at corresponding developmental stages in the 2 species. This suggests that close proximity to LTi cells may support the survival or maturation of later-stage B cell populations, as these subsets were absent in RORγt^KO^ mice, which lack functional LTi cells.

Our data also implicate LTi-mediated signaling through the LT-beta receptor (LTBR) on B cells as a potential mechanism for this interaction. LTBR expression was consistently higher on all intestinal B cell subsets compared with fetal liver B cells. Additionally, we observed colocalization of human B cells with BAFFR, and computational analysis predicted interactions between B cells and LTi cells via this receptor. Notably, this BAFFR-mediated interaction appeared to be unique to human fetal intestine, as BAFFR expression on mouse intestinal B cells was not definitively detected.

A previous study identified local receptor recombination in neonatal mouse intestines in response to microbial colonization, thus shifting our understanding of how a local B cell repertoire is established (6). To our knowledge, this is the first study to reveal that mouse intestinal B cells express *Rag* during gestation. These findings indicate that B cells are actively recombining in the developing intestines, a process that may be affected by a variety of cell intrinsic or extrinsic factors in utero. This suggests that B cells in the developing intestine may undergo selection for self-antigens, potentially serving as local, homeostatic B cell population during early life. Our findings highlight an axis and time for the establishment of a preimmune repertoire in the absence of microbial signals. This reveals a previously unrecognized window and location for the establishment of a preimmune B cell repertoire, independent of microbial stimulation. Juxtaposed with the neonatal intestinal immune repertoire, we hypothesize that these developing B cells may contribute to shaping early-life immune homeostasis.

There are various limitations to our study. There are no other known genetic models of LTi deficiency aside from the RORγt-deficient mice, which also lack Th17 cells (55). Th17 cells are an essential T cell subset in the gut through their production of IL-17 among other cytokines to regulate intestinal inflammation and immune defense (55). However, due to this, the postnatal effect of LTi-deficiency on the intestinal B cell compartment is confounded by the absence of Th17 cells, which are known to cause intestinal inflammation and dysbiosis (56). However, Th17 cells are incredibly rare if at all present prenatally and, therefore, are likely unrelated to the phenotype we observe at this time point. The effect of cell-specific loss of IL-7, TSLP, CXCL12 or other related cytokines on B cell development in fetal intestines could not be evaluated here due to the lack of available genetic models. Furthermore, due to the size of the mouse intestinal tissue at embryonic stages, we did not distinguish between small and large intestinal tissue in mice. This limitation impaired our ability to accurately identify which region of the intestinal tract is essential for B cell development in mice and to determine whether this is happening in proximal or distal anatomical regions. However, our human data are specific to the SIs, and considering that PPs form primarily in the SI, we suspect that this is the primary anatomical region implicated in this process (57).

Collectively, our findings reveal a pathway for B cell development in the fetal intestine and highlight LTI cells as crucial regulators of this process. We suggest that disruptions in this pathway could have major consequences for embryonic intestinal immunity. Furthermore, our study underscores the need to explore other mucosal and nonmucosal tissues as unique and preferential developmental niches for immune cells, specifically B cells, during embryogenesis. Finally, the mechanisms we uncover that underlie tissue-specific B cell lymphopoiesis pave the way for future research into the relevance of these unique developmental niches. One key question that emerges is how the embryonic intestinal microenvironment and B cell development is affected by maternal stressors and infections and its subsequent effect on shaping protective immune responses or a predisposition to pathological inflammation.

## Methods

### Sex as a biological variable.

By nature of this study, each experiment was conducted independent of sex in both mice and human samples, thus the data represented here is reported as combined male and female. For mouse studies, this is due to the nature of collecting and pooling intestines from litters for assays that require increased cell yield (i.e. flow cytometry).

### Human scRNA-Seq data analysis.

The human scRNA-Seq data set was previously published (19). The fetal samples we used for this study: first trimester (8–13 weeks gestational age) *n* = 14, second trimester (14–23 weeks gestational age), *n* = 13. However, the B cell related data was reanalyzed for the current manuscript. The fetal B cells were clustered using Scanpy (v1.9.2) package (58) as described in Gu et al. (19). Briefly, gene expression in each cell was normalized and log-transformed. Afterward, highly variable genes were identified using the scanpy.pp.highly_variable_genes function with default parameters. In addition, the effects of the percentage of mitochondrial genes, percentage of ribosomal protein genes, and unique molecular identifier (UMI) counts were regressed out using scanpy.pp.regress_out function before scaling the data. Batch correction of samples was performed with bbknn (v1.5.1) (59). Dimensionality reduction and Leiden clustering was carried out on the remaining highly variable genes, and the cells were visualized using UMAP plots. Cell types were manually annotated based on known markers genes found in the literature. Developmental trajectory analysis was performed using PHATE (v1.0.10) (60). The normalized datasets were imported into PHATE to instantiate a PHATE estimator object with default parameters.

### In situ hybridization (RNAscope) combined with IF of human intestinal tissue.

The RNAscope Multiplex Fluorescent Reagent Kit v2 with TSA Vivid Dyes assays (Advanced Cell Diagnostics) were conducted using target RNA probes, IL-7 (#424251), CXCL12 (#422991), IL-7R (#421241), CXCR4 (#310511), CD34 (#560821), RAG1 (#515171), CD19 (#402718), TNFSF13B (#406971), and RORC (#311061) based on the manufacturer’s instructions. In brief, paraffin sections were deparaffinized, treated with a citrate buffer, and hybridized sequentially with target probes. After RNA signal development, a blocking step was performed with 10% horse serum, followed by overnight incubation at 4°C with desired primary antibodies (LYVE1, Biotechne, #AF2089 ; CD31, Cell Signaling Technology, #3528) dilution, and incubated with secondary antibody. Images were taken at 10x or 20x using Echo Revolve microscope. RNA signals appear as dots. The distance between RAG1+ cells and the nearest LYVE1+ cells were quantified using Fiji ImageJ (NIH).

### Mouse samples and breeding.

C57BL/6J (WT) and Rorctm1Litt/J (RORγt^KO^) (ISMR_JAX: 007571) mice were purchased from Jackson Laboratory. Mice were quarantined, then bred in animal facilities at Tufts University, Boston, MA, USA. The RORγt^KO^ colony was maintained using a heterozygous RORγt (RORγt^HET^) breeding strategy (RORγt^HET^ x RORγt^HET^) to avoid breeding depression. Three to 5 mice were housed in cages in a pathogen-controlled environment with unrestricted access to water and food. The vivarium was maintained in a 14h light/10h dark cycle, at an ambient temperature of 70-74 deg. F, with humidity between 30-70% RH. All experiments were performed according to protocols approved by the Institution of Animal Care and Use Committee (IACUC) at Tufts University and Tufts Medical Center (protocol no. B2023-30). Male and female mice used for experimental breedings were aged between 8 and 20 weeks. For embryonic time points, male and female mice were cohoused until detection of a vaginal plug (E0.5) then separated. For embryonic time points, experiments were conducted according to the date of separation in a controlled breeding procedure. Experiments with embryos from RORγt^KO^ x RORγt^HET^ breedings were blinded and individual samples were labeled with numbers during the time of experiment and genotyped following completion.

### scRNA-Seq analysis of MCDA.

Publicly available scRNA-Seq data from intestinal tissue at E14.5 (GSE176063) were downloaded as raw count files and converted into Seurat objects in R. These files were integrated using RunHarmony (61) and normalized according to standard parameters (mitochondrial & ribosomal genes < 20%, nFeatures > 200, nCount > 500). Cells were clustered using 25 principal components and resolution was set for 1.0. Preliminary identification of cell populations was conducted using a combination of scType and manual annotation based on top differentially expressed genes.

### scRNA-Seq predictive signaling analysis.

Cellchat predictive analysis was completed on the mouse and human scRNA-Seq datasets using the respective ligand-receptor pair databases in each species according to previous publications (39, 62). Briefly, a CellChat object was created for each group from the total intestines Seurat object by running createCellChat() and a CellChatDB.mouse or CellChatDB.human dataset was referenced for putative signaling networks. Over or under expressed ligands and receptors were predicted by running the identifyOverExpressedGenes() function. The computeCommunProbPathway() function was used on the updated objects to identify specific pathways that were upregulated with a truncated mean method using a trim = 0.01. Cumulative outgoing (ligand) and incoming (receptor) pathways were visualized using netAnalysis_signalingRole_heatmap() function and specific pathways to identify senders and receivers were visualized using netVisual_circle().

### Single-cell isolation from mouse fetal tissues.

Pregnant mice were euthanized by CO_2_ asphyxiation followed by cervical dislocation according to IACUC protocols (Tufts University and Tufts Medical Center, protocol no. B2023-30) and procedures. Embryos were carefully removed, decapitated, and stored in ice cold 1X phosphate buffered saline (PBS) while isolating the fetal liver and intestines. Intestines were processed using mechanical and enzymatic digestion. Briefly, tissues were chopped and processed using a Small Intestine Digestion Kit (Miltenyi), samples were centrifuged and cells were counted using Countess (Invitrogen). For embryonic time points with WT and RORγt^KO^ mice, 4–8 mice were pooled according to the time point, as listed in the figure legends.

### Antibody staining and flow cytometry of mouse tissue single cell suspensions.

Samples were split and stained with 2 separated antibody panels to detect distinct cell populations. To detect LTis and hematopoietic progenitor populations, cells were incubated with Live/Dead stain (BioLegend, 428105) for 20 minutes at 4C in the dark. Cells were washed, then incubated with Fc block (BioLegend, CD16/32) for 20 minutes, then incubated with an antibody mixture containing B220, SCA1, NKp46, CD196, CD3e, CD8, LY6G, LY6C, NK1.1, CD45, CD19, CD135, IgM, IL-7R, a4b7, CD4, and CD117 (Supplemental Table 1) for 20 minutes at 4C. The same process was used to stain and detect B cells, however cells were stained with a different Live/Dead stain (Invitrogen, L34957) and an antibody mixture containing CD5, CD103, B220, CD20, CD23, CD69, IgD, CD43, CD45, CD138, CD93 (AA4.1), CD22, and CD19. All samples were run and data were collected on a Cytek Aurora 5-laser cytometer.

### IF staining of mouse tissue sections.

OCT cryopreserved tissues were cut into 10 μM sections using a Leica Cryotome and stored at –80°C until staining. OCT was removed by washing slides with 1x PBS and samples were fixed by incubating with 4% PFA for 20 minutes at room temperature. Samples were washed then blocked and permeabilized with 5% donkey serum in 0.1% Triton for 1 hour at room temperature. Unconjugated antibodies to RORγt or LYVE1 were added, and samples were incubated overnight at 4°C. Secondary antibodies (Supplemental Table 1) were added after washing and incubated for 6 hours at 4°C. Conjugated antibody to B220-FITC (Supplemental Table 1) were added after washing and samples were incubated overnight at 4°C. After a final wash, hoescht (Thermo, 62249) was added to each sample for 3 minutes, removed, then slides were coverslipped with fluormount (Southern Biotech, 0100-01) and imaged using a Leica SP8 confocal microscope with a Leica DMi8 base.

### In situ hybridization (RNAscope) of mouse intestinal tissue.

The RNAscope Multiplex Fluorescent Reagent Kit v2 with TSA Vivid Dyes assays (Advanced Cell Diagnostics) were conducted using target RNA probes, CD19 (#314711), RAG (#545131-C2), RORC (#403661-C3), based on the manufacturer’s instructions. In brief, frozen sections were fixed in 10% Formalin, treated with a citrate buffer, and hybridized sequentially with target probes. Images were taken at 43x using LEICA SP8 confocal microscope. RNA signals appear as dots.

### Statistics.

Statistical comparisons between 2 groups were performed using a 2-tailed Student’s *t* test. For comparisons involving more than 2 groups, 1-way or 2-way ANOVA was applied as appropriate, followed by post hoc multiple comparison tests (Bonferroni or Dunnett). Details of the statistical tests used, along with corresponding *P* values, means, medians, and SEM, are provided in the figures and figure legends. Data are shown as mean ± SEM. *P* < 0.05 was considered statistically significant.

### Study approval.

We combined data collected from human intestinal tissue obtained from patients undergoing elective termination of pregnancy with IRB approval and informed consent. Considering the exploratory nature of this study, power calculations were not completed prior to the inception of the study. The number of mice for each experimental group was kept between 3 and 10 mice for statistically significant conclusions. All experiments were performed in accountancy with the Tufts University IACUC. All murine samples collected for IF and flow cytometry were used in the analysis without exclusion. Sample numbers and *n* for each experimental group are listed in the figure legends on a per experiment basis, and all individual values are present within the respective figures.

### Data availability.

All data associated with this study are included in the paper and supplementary information. Human and mouse scRNA-Seq data are referenced where applicable from previously published studies (19, 36). Values for all data points in graphs are reported in Supporting Data Values file. Additional information may be available upon request.

## Author contributions

Conceptualization was contributed by KAC, WG, LK, and SS. Methodology was contributed by KAC, WG, LK, and SS. Investigation was contributed by KAC, WG, LP, EGS, WW, and GCT. Visualization was contributed by KAC, WG, and LP. Funding acquisition was contributed by KAC, SS, and LK. Supervision was contributed by SS and LK. Writing of the original draft was contributed by KAC, WG, LK, and SS. Review and editing were contributed by KAC, WG, LK, and SS. KAC and WG are listed as co–first authors. Both authors contributed experimentally and conceptually in equal parts. KAC is listed first as she generated primary drafts of general sections; however, WG contributed extensively for review and revision of subsequent iterations, granting them equal first author status, but listing KAC first.

## Supplementary Material

Supplemental data

Supplemental table 1

Supporting data values

## Version 1 09/02/2025

In-Press Preview

## Version 2 10/08/2025

Electronic publication
