# Supplementary material for: Conserved interactions with stromal and immune cells coordinate de novo B cell lymphopoiesis in fetal intestines
Source: JCI Insight. 2025 Sep 2;10(19):e192550. doi: 10.1172/jci.insight.192550 (PMC12513481; doi:10.1172/jci.insight.192550)
Supplement: Supplemental data [file jciinsight-10-192550-s196.pdf]

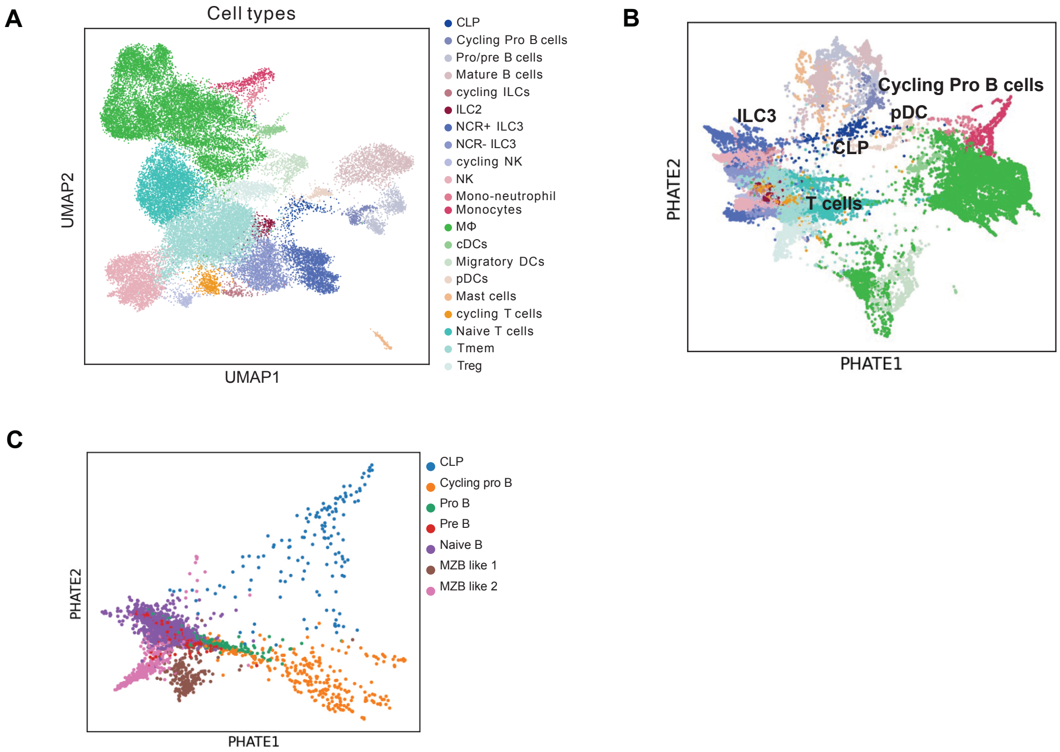

Supplemental Figure S1. Human scRNA sequencing B cell PHATE analysis. (A) UMAP visualization of major fetal immune cells. PHATE visualization colored by total fetal immune cell types **(B)** and fetal B cells **(C)**.

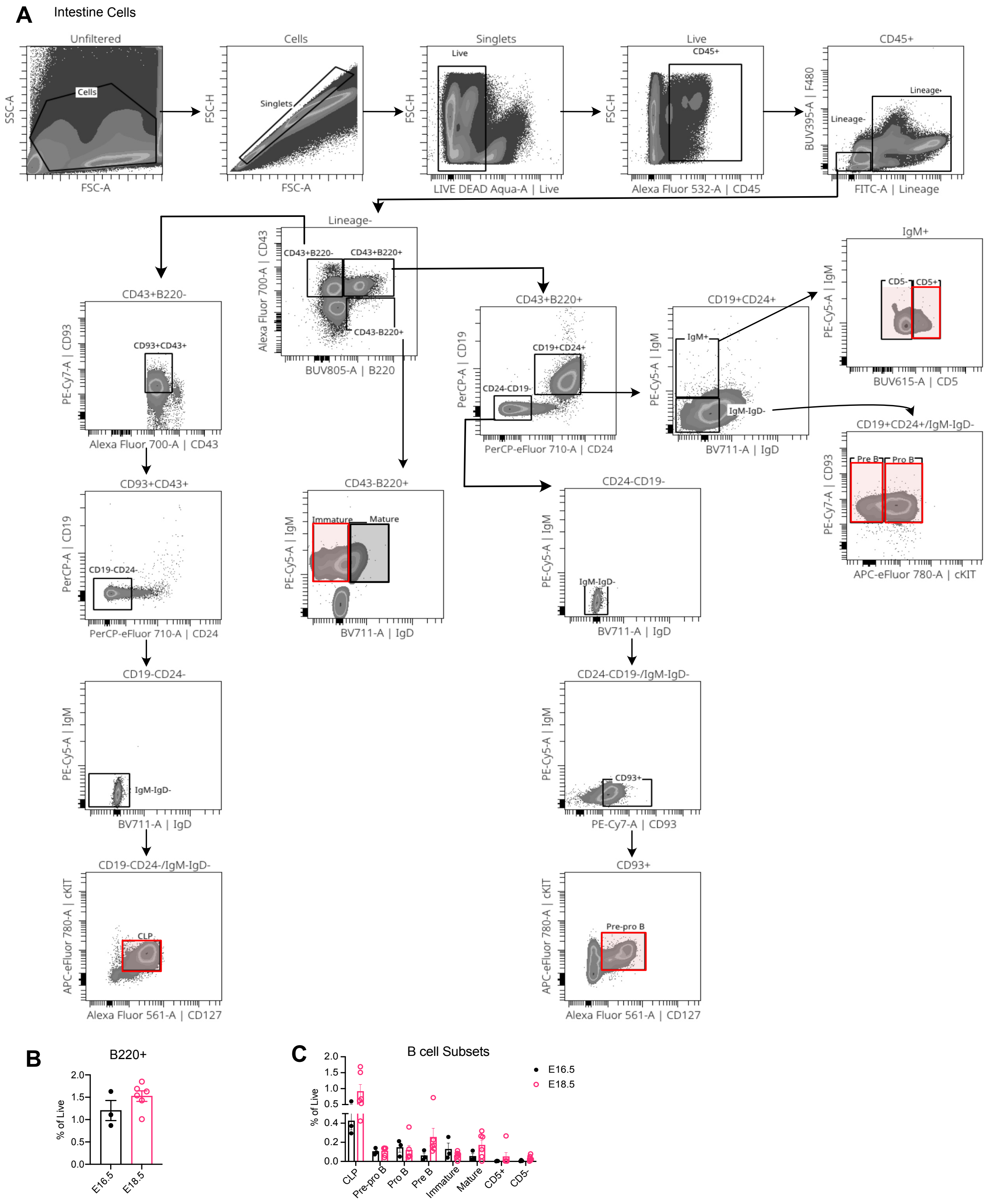

Supplemental Figure S2. Gating strategy and quantification of intestinal B cell subsets. (A) Gating strategy for identification of B cell subsets from fetal (E16.5) intestinal tissue of WT mice. Lineage gate includes CD3e, CD8, LY6G, LY6C, NK1.1, & Ter119 **(B)** Percentage of B cell subsets at E16.5 with SEM (n=3).

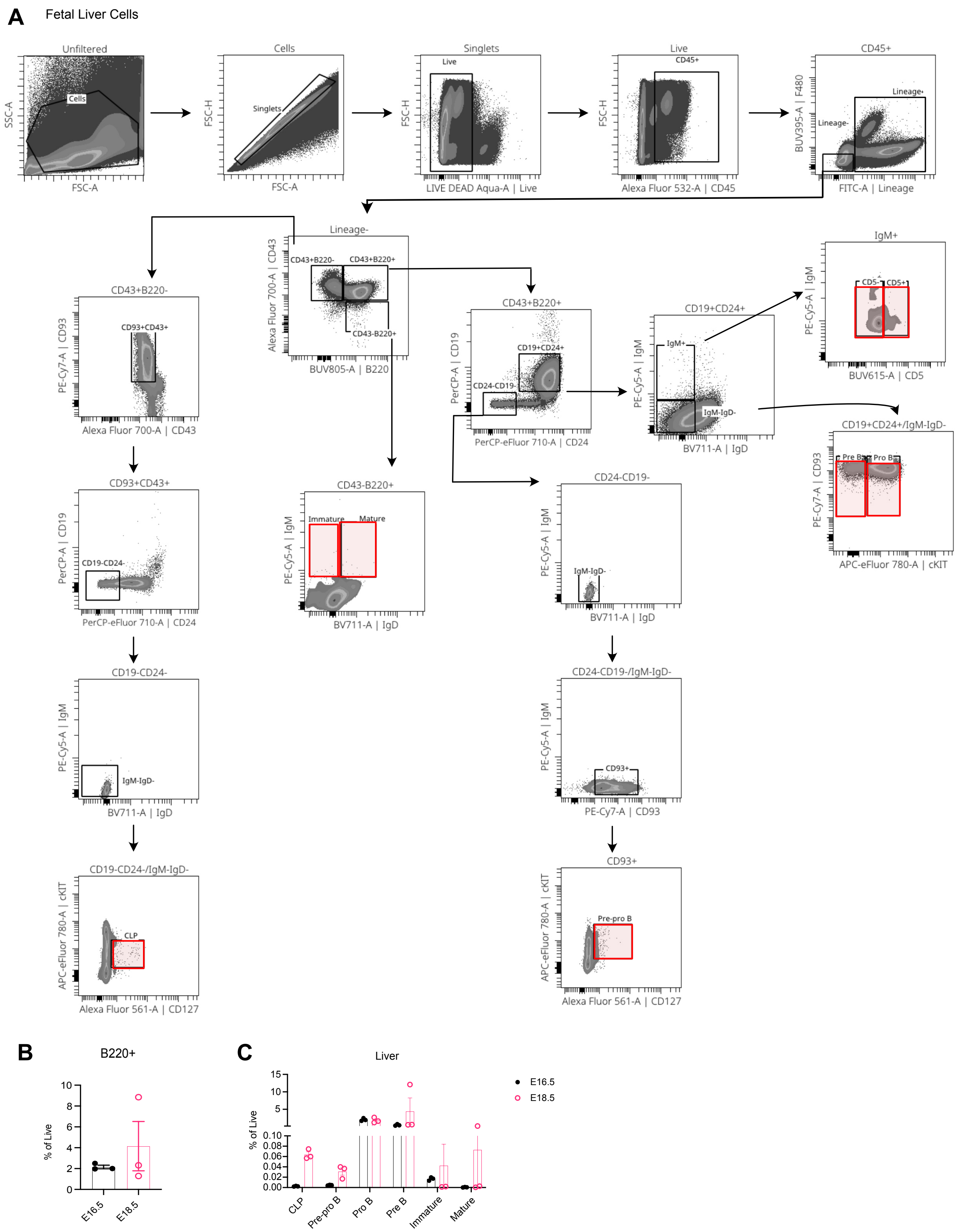

Supplemental Figure S3. Gating strategy and quantification of fetal liver B cell subsets. (A) Gating strategy for identification of B cell subsets from fetal (E16.5) intestinal tissue of WT mice.

Lineage gate includes CD3e, CD8, LY6G, LY6C, NK1.1, & Ter119 **(B)** Percentage of B cell subsets at E16.5 with SEM (n=3).

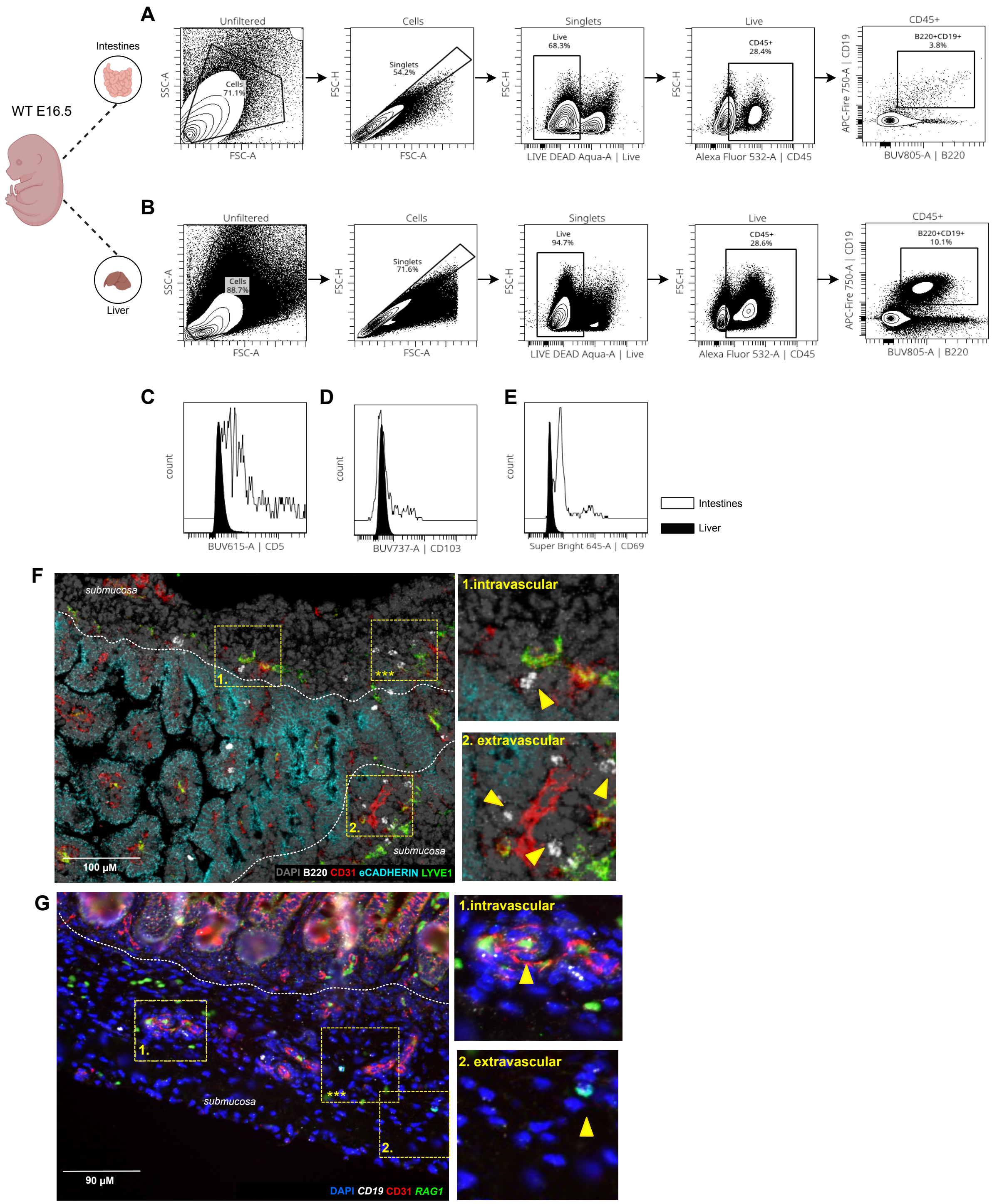

Supplemental Figure S4. Characterization of B cell tissue residency and distribution in fetal mouse intestines. Gating strategy for total B cells (B220^{+}CD19^{+}) in intestines **(A)** and liver **(B)** at E16.5. Representative histograms to depict expression of CD5 **(C),** CD103 **(D)** or CD5 **(E)** on B cells in fetal liver and intestines (E16.5), n=3. **(F)** Extended representative image of mouse fetal intestinal tissue (E18.5) to identify B cells with quantification of B cells that were intravascular, based on direct colocalization with CD31 (B220^{+}CD31^{+}), or extravascular, (B220^{+}CD31^{-}) **(G)**

Extended representative image of human fetal intestinal tissue with IF staining CD31 (red) and probed with *RAG1* (green) and *CD19* (white), nuclei stained with DAPI (blue) to identify B cells that were intravascular, based on direct colocalization with CD31 ($CD19^{+}RAG1^{+}$CD31^{+}), or extravascular, ($CD19^{+}RAG1^{+}$CD31^{-}).

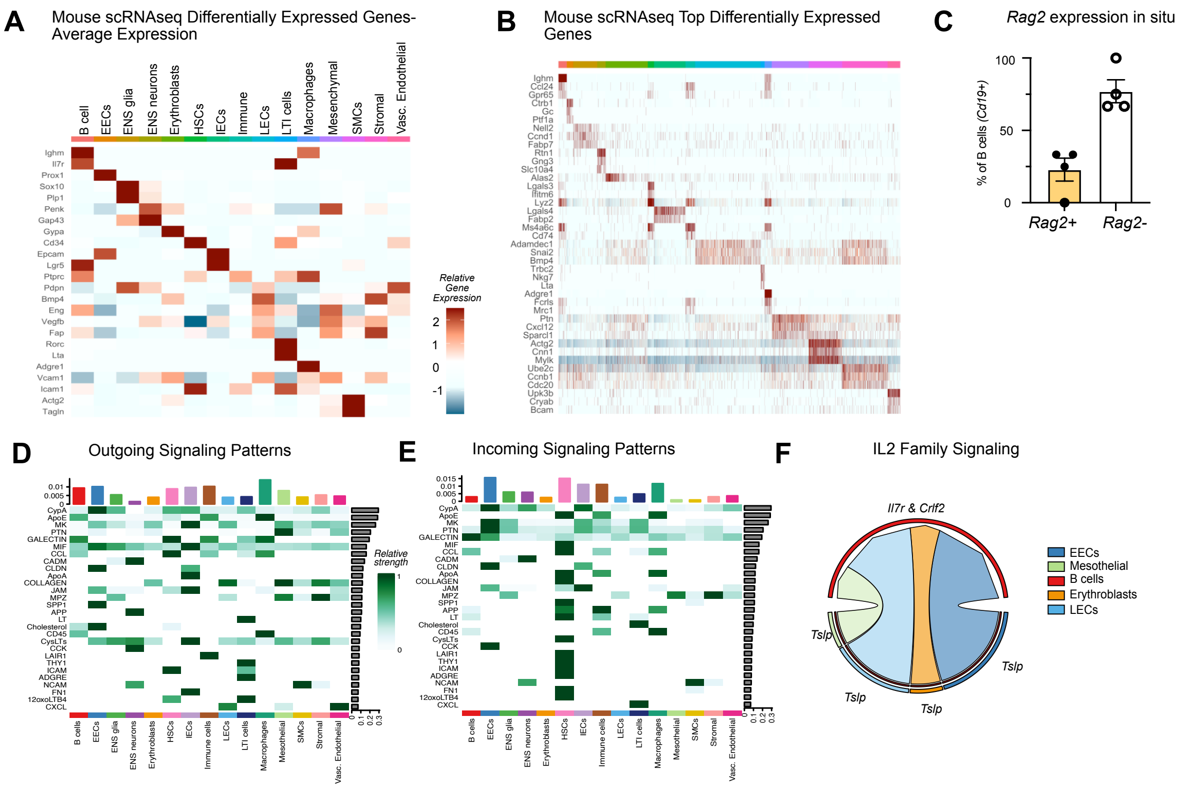

Supplemental Figure S5. Mouse scRNA B cell and stromal cell predicted interactions for receptor recombination. (A) Heatmap of top 3 differentially expressed genes within each cluster. Top differentially expressed genes were used for the computational algorithm, ScType, to determine populations. **(B)** Heatmap of average expression of curated genes for population identification by scRNA sequencing of E16.5 mouse tissue. **(C)** Quantification of *Rag2*$^{+}$ and *Rag2*$^{-}$ B cells (*Cd19+*) by RNAscope in intestinal tissue (E18.5) (n=4 mice). Heatmaps of top outgoing **(D)** and incoming **(E)** signaling pathways as predicted by CellChat. Top colored bars correspond to the cumulative incoming or receiving status of distinct cell populations labeled at the bottom. Gray bars correspond to the relative strength/enrichment of the signaling pathway across all clusters. **(F)** Chord diagram depicting the ligand:receptor signaling within the IL2 family network between E14.5 cell populations. Arrow color corresponds to sender population and arrow head direction with the receiver (outer ring color).

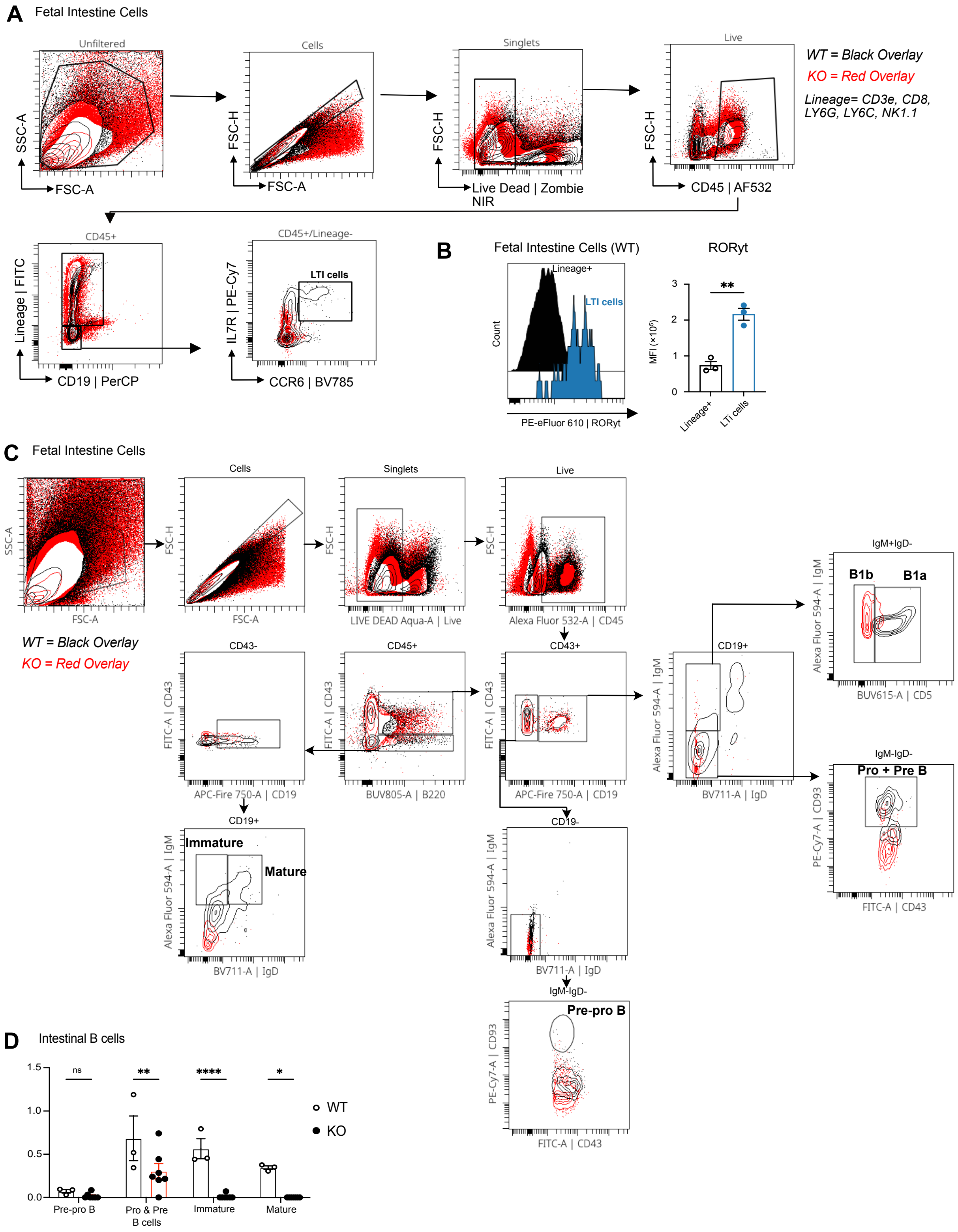

Supplemental Figure S6. Comparison of B cell subsets in WT and RORyT^{KO} fetal mouse intestines. (A) Mouse intestinal cells (E16.5) from WT (black) and RORyt^{KO} mice (red) overlaid in a gating strategy for identification of LTi cells. Lineage gate defined in a key. **(B)** Expression

of RORyt in WT mice by intracellular staining depicted in a histogram in LTi cells (blue) and Lineage+ cells (black). MFI quantified between WT Lineage+ and LTi cells ($n = 3$, Student's t test, $p < 0.01$ = **, with SEM). **(C)** Mouse intestinal cells (E16.5) from WT (black) and $RORyt^{KO}$ mice (red) overlaid in a gating strategy for identification of B cell subsets by flow cytometry. **(D)** Quantification of B cell subsets in WT and $RORyt^{KO}$ mice($n = 3$, two-way ANOVA, $p > 0.05$ = ns, <0.05 = *, <0.01 = **, & <0.0001 = ****).

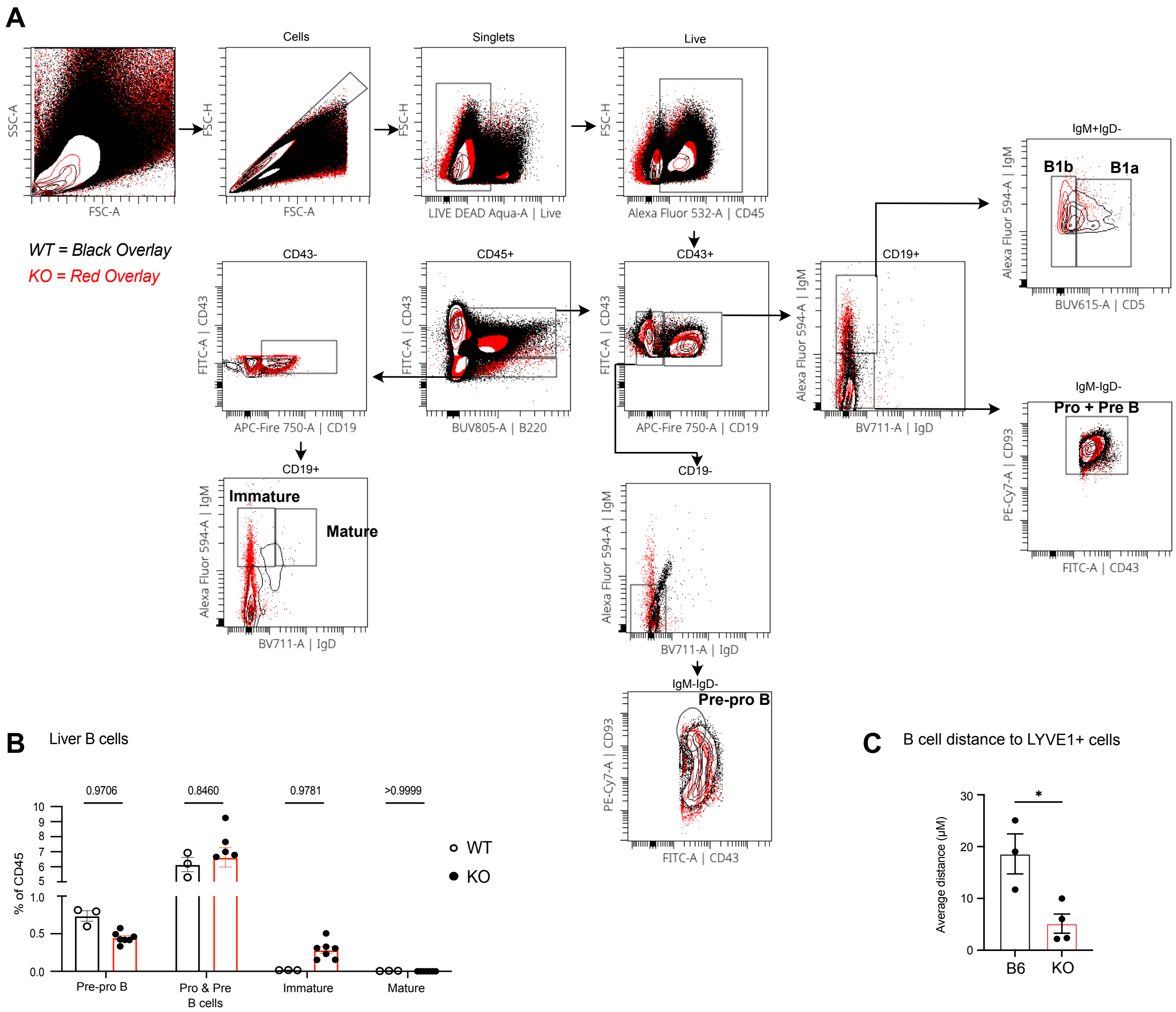

Supplemental Figure S7. Comparison of B cell subsets in WT and RORγT^{KO} fetal liver and tissue distribution. (A) Mouse liver cells (E16.5) from WT (black) and RORγT^{KO} mice (red) overlaid in a gating strategy for identification of B cell subsets by flow cytometry. **(B)** Quantification of B cell subsets in WT and RORγT^{KO} mice (n = 3, two-way ANOVA, p-values reported with SEM). **(C)** Quantification of average distance between LYVE1^{+} cells and B220^{+} cells in IF images of WT and RORγT^{KO} mice (E18.5) (n=3 mice, Student's t test, $p < 0.05$ = * with SEM)

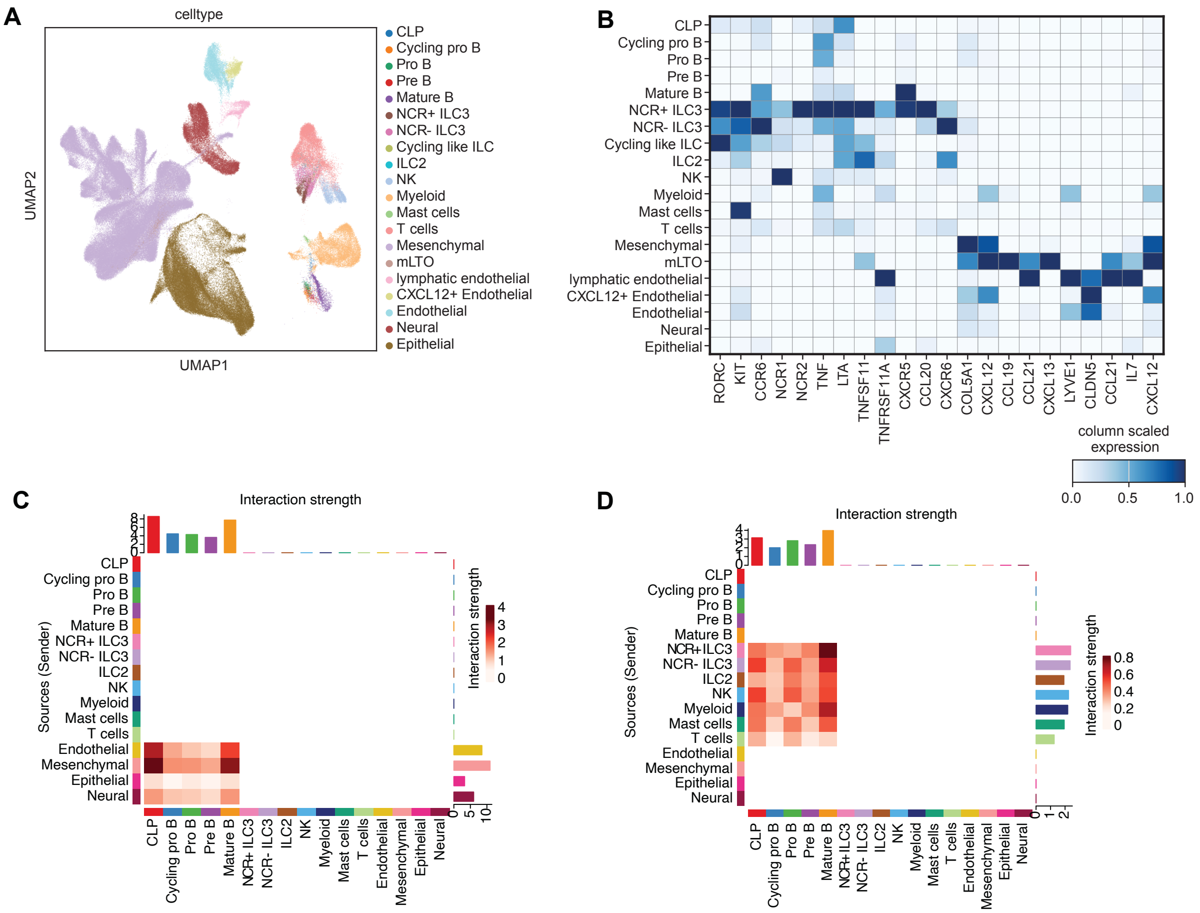

Supplemental Figure S8. Human scRNA sequencing background and predicted signaling. (A) UMAP visualization of fetal major cell types**. (B)** Heatmap showing scaled mean expression of selected marker genes, used to identify the major cell types in **A**. Heatmap of cell-cell interaction strengths between receptor signaling in B cell subsets (x axis) and target signaling in non-immune cells (y axis) **(C)** and immune cells (y axis) **(D)**.

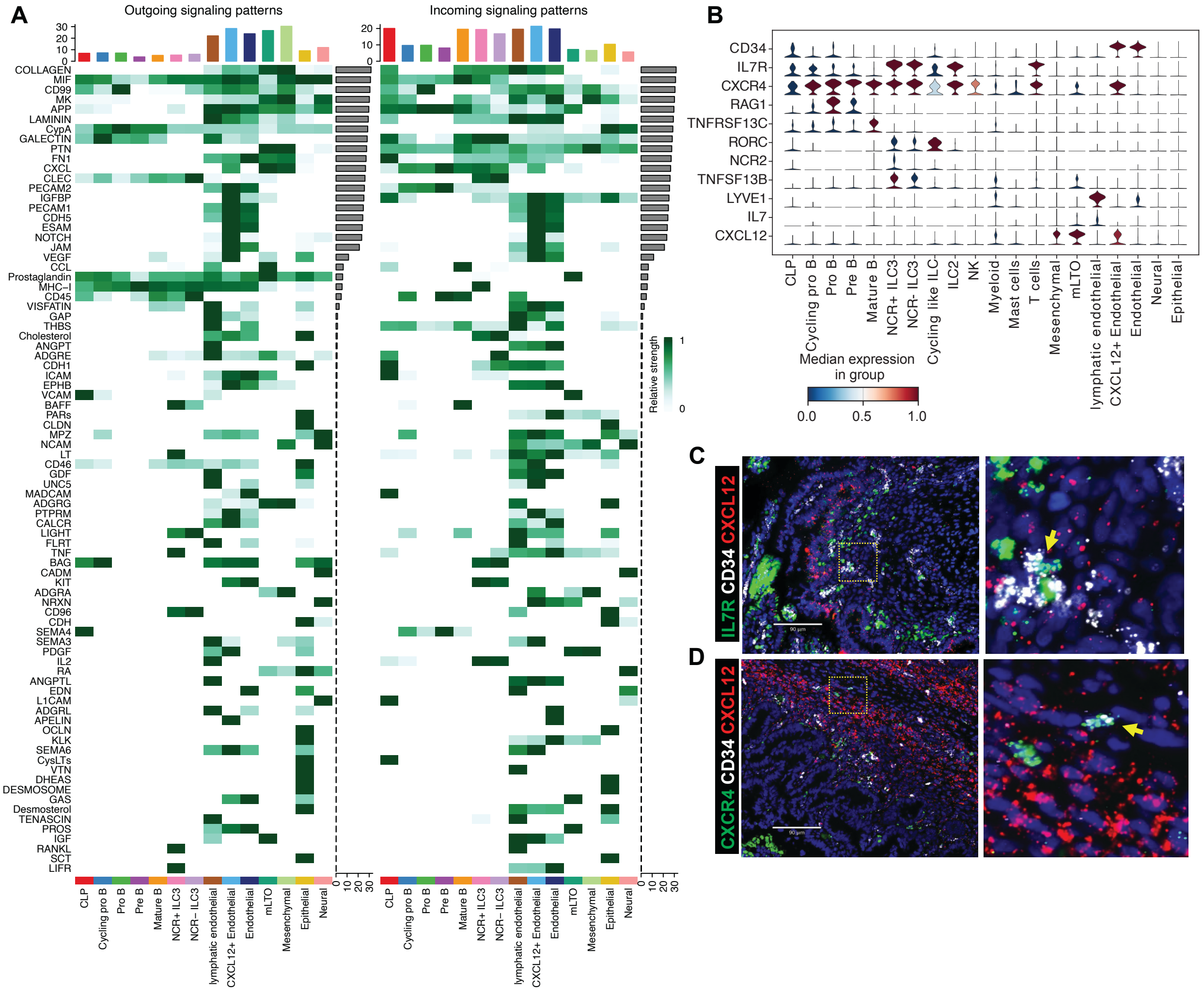

Supplemental Figure S9. Human predicted signaling networks, scRNAseq and immunofluorescence cell identification. (A) Heatmaps showing the relative strength of the signaling pathways among B cells, ILCs and non-immune cells in the outgoing and incoming signaling patterns. **(B)** Stacked violin plot of selected gene expression used in the validation of various populations with RNAscope. **(C)** Representative multiplex RNAscope images of fetal small intestine (21 weeks gestational age). The yellow square highlights the region magnified on the right. Yellow arrows represent precursor B cells (CD34+IL7R+). Scale bar represents 90 µm. **(D)** Representative multiplex RNAscope images of fetal SI (21 weeks gestational age). The yellow square highlights the region magnified on the right. Yellow arrows represent precursor B cells (CD34+CXCR4+). Scale bar represents 90 µm.
